# Connectivity-Rewired Construction of Hydrogen-Bonded Azo-Macrocycles Enables Photoswitchable Recognition of Lithium Ions

**DOI:** 10.3390/molecules31071086

**Published:** 2026-03-26

**Authors:** Chengyu Tan, Kuirong Fu, Zhiyao Yang, Song Qin, Yimin Cai, Wen Feng, Xiaowei Li, Lihua Yuan

**Affiliations:** College of Chemistry, Key Laboratory of Radiation Physics and Technology of Ministry of Education, Institute of Nuclear Science and Technology, Sichuan University, Chengdu 610064, China; chengyutan2001@163.com (C.T.); frederrean@163.com (K.F.); yangzhiyaohaha@sina.com (Z.Y.); qinsong@scu.edu.cn (S.Q.); ymcai@scu.edu.cn (Y.C.); wfeng9510@scu.edu.cn (W.F.)

**Keywords:** photoresponsive recognition, hydrogen-bonded azo-macrocycles, lithium cation, ion receptor

## Abstract

Photoresponsive hydrogen-bonded azo-macrocycles capable of selectively recognizing lithium cation were constructed by reversing the amide–azobenzene connectivity, which redistributes electron density and preorganizes four carbonyl oxygen donors into a smaller, more convergent cavity. Compared with a connectivity-isomeric reference macrocycle, the new receptor displays a pronounced preference for Li^+^, in which complexation with LiClO_4_ shows a slow exchange on the ^1^H NMR timescale and an association constant (*K_a_*) exceeding 10^4^ M^−1^, whereas the reference binds Li^+^ weakly (<5 M^−1^). In contrast, both hosts exhibit only modest binding toward Na^+^ (10^2^~10^3^ M^−1^) and fast exchange, consistent with size/geometry matching of the compressed cavity to Li^+^. The newly designed azo-macrocycles reveal a highly selective recognition of Li^+^ thanks to the more evenly arrayed four amide oxygens enclosing a cavity of small dimension. Notably, *E*/*Z* photoisomerization of macrocycle switches the binding regime, enabling reversibly light-triggered Li^+^ binding under UV irradiation and recapture under visible light. This work establishes a new photoresponsive receptor based on H-bonded azo-macrocycles for photoswitchable recognition of Li^+^.

## 1. Introduction

Hydrogen-bonded aromatic oligoamide macrocycles [1,2,3] (H-bonded aramide macrocycles for short) were first discovered during the synthesis of folded structures containing intramolecular hydrogen bonds [4,5,6,7,8]. These macrocyclic compounds feature a 2D planar conformation with a cavity decorated with amide functionalities pointing inwards, which enable them to present a plethora of rich host–guest chemistry [9], leading to diversified applications, including selective molecular recognition [10,11,12,13], catalysis [14], liquid crystals [15], rotaxane [16] and molecular machines [17]. Among them, H-bonded aramide macrocycles of the smaller size are intriguing as they are able to bind smaller alkali metal ions [18]. However, these macrocycles have not shown the responsive behavior until recently, when the introduction of photoresponsive groups into an H-bonded aramide macrocycle produces an artificial system capable of photo-controlled recognition of organic guests and photoresponsive artificial molecular machinery [19]. Endowing macrocycles with photo-switchable properties is appealing to supramolecular chemists on account of their abilities in photo-controlled switching of H–G complexation [20,21,22,23,24,25,26]. This is particularly attractive for use in providing desired chemical functions, such as mimicking light-driven ion transport through membranes [27,28] and photo-controlled catalysis [29,30]. In terms of macrocyclic structures, most studies on photo-controlled cation binding focus on photoresponsive crown ether-based receptors with incorporated azobenzene residues [31,32,33]. Pursuing various types of light-sensitive macrocycles with efficient and selective binding ability has invariably been a charming goal to enrich the toolbox of photoresponsive macrocycles and find potential applications for use in practice. In this regard, H-bonded aramide macrocycles are expected to be one of the options for this goal. So far, macrocyclic receptors capable of photoresponsive capture of alkali metal cations remain very rare [34].

**Figure 1:**
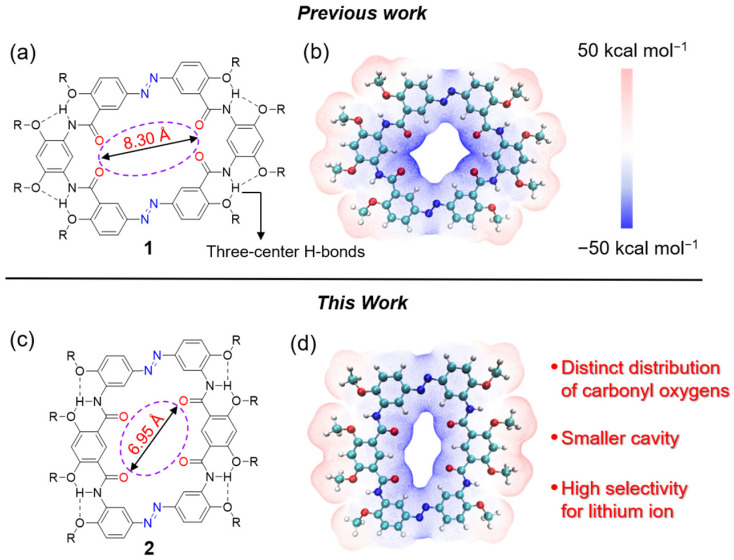
Hydrogen-bonded azo-macrocycles. (**a**) Chemical structure of macrocycle **1** (previous work) and (**b**) its optimized geometry and mapped surface electrostatic potential (ESP). (**c**) Chemical structure of macrocycle **2** (this work) and (**d**) its optimized geometry and mapped ESP, highlighting the smaller, more convergent cavity. In the ESP maps, red denotes regions of more positive potential (electron-poor), whereas blue denotes regions of more negative potential (electron-rich). Geometries were optimized at the B3LYP/6-31G(d) level with the exterior side chains replaced by methyl groups.

Our previous work on photoresponsive H-bonded receptors (Figure 1a) shows selective complexation of organic cations along with accurate release and capture of guest molecules under photo-controlled conditions. Preliminary experiments with this macrocycle revealed recognition of lithium ions and sodium, but exhibited low affinity for Li^+^ and a fast exchange between the macrocycle and Li^+^ on the NMR time scale, which precludes the receptor system from capturing and releasing the cation in an accurately controlled fashion. Accessing a slow host–guest exchange of complexation on the NMR time scale in designed receptors with azobenzene units is crucial to realize reversibly light-triggered binding of guests by photo-isomerization [19]. In this regard, photoresponsive recognition of metal ions has found widespread applications in industry and biology [35,36,37,38,39]. Close inspection of the macrocyclic structure of azo-macrocycle **1** by molecular modeling indicates that four amide oxygen atoms are widely distributed with a less populated electron-rich area (Figure 1b) compared to our prior reported receptor that exhibits excellent selectivity for lithium ions [18]. We speculated that creating a more concentrated electron-rich area in the cavity by altering the way in which electron-donating groups are arrayed would improve the binding affinity for alkali metal ions and produce a receptor system with a slow exchange of complexation.

To this end, a new receptor is created in which four amide oxygen atoms, well preorganized by three-center hydrogen bonds, are placed close to each other to form a smaller cavity (6.69 × 7.69 Å) as indicated by DFT computation (Figure 1c and Figure S43). Here we report the design and synthesis of photoresponsive H-bonded azo-macrocycles and reversibly photoresponsive binding of Li^+^. Instead of connecting two azobenzene units to amide carbonyl groups, the macrocyclic construction proceeds the other way round from the two photoactive units tethered to amide NH groups. The distance between two latitudinal amide oxygen atoms becomes shorter. Such a design endows the binding pocket with more dense electron donors as indicated by surface electrostatic potentials prediction (Figure 1d), and thus would enable stronger association with such metal ions of smaller diameter as lithium ions. As expected, the binding ability for lithium is significantly enhanced with azo-macrocycle **2**, along with the characteristic slow exchange of complexation. Furthermore, reversibly photoresponsive binding of lithium ions is achieved using light as a stimulus through changing the shape and geometry of the macrocyclic framework. Selective recognition of lithium cations is intriguing (in demand) owing to its extensive applications in modern battery technology, glass and ceramics processing, lubricants, and pharmaceuticals [40,41,42,43,44].

## 2. Results and Discussion

**Scheme 1:**
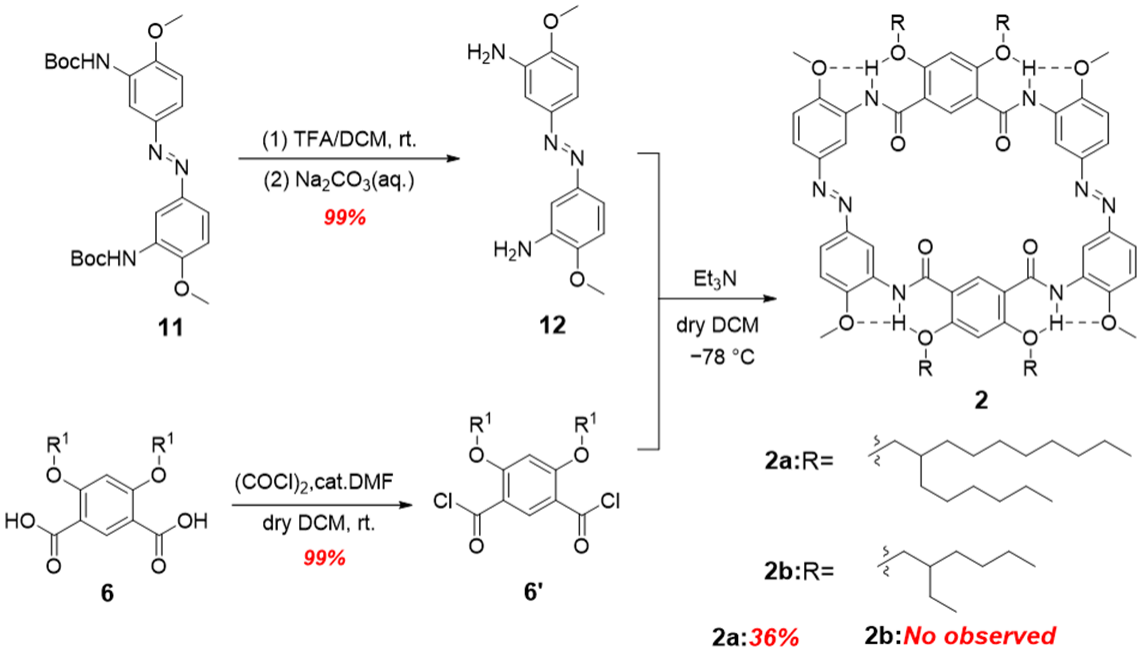
Synthesis of hydrogen-bonded azo-macrocycles **2** via a [1 + 1] macrocyclization approach. The dashed lines indicate the presence of intramolecular hydrogen bonds in the chemical structures.

**Scheme 2:**
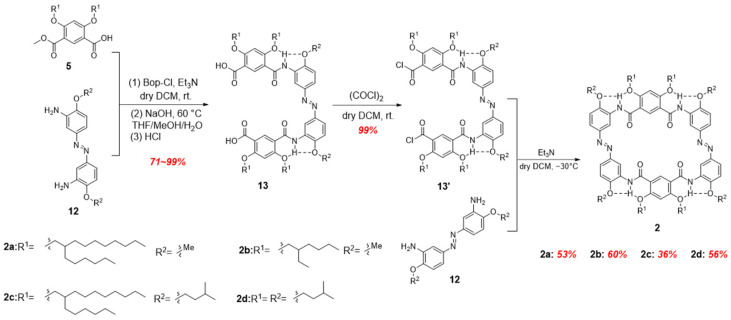
Synthesis of hydrogen-bonded azo-macrocycles **2** via a [4 + 1] coupling approach. The dashed lines indicate the presence of intramolecular hydrogen bonds in the chemical structures.

Different from traditional approaches for preparing macrocycles such as high dilution [45,46], template [47,48,49], dynamic covalent [50,51,52] macrocyclization which rely on external factors, H-bonded macrocycles are built upon the use of internal factors [8,53], i.e., taking advantage of a crescent and rigid backbone of oligomeric precursors enforced through intramolecular hydrogen bonds [54]. The selection of appropriate building units that are able to hydrogen bond adjacent units is crucial to the orientation and preorganization of amide linkages in resultant shape-persistent [55] H-bonded macrocycles. Thus, to change the distribution of electron-rich area in the cavity, a diamino-substituted diazobenzene derivative **12** installed with two ortho-diamino groups rather than ortho-dicarboxy groups is employed to react with dialkoxy-substituted isophthaloyl dichloride **6**′ (Scheme 1). The preparation of **12** was accomplished via Boc-protected precursor **11**. The condensation reaction via one-pot cyclization procedure was carried out at very low temperature (−78 °C) since lower temperatures are generally favorable to such hydrogen bonds-assisted macrocyclization [8], particularly in the case when the macrocyclic backbone is not fully rigidified by intramolecular hydrogen bonds. The desired H-bonded aramide macrocycle is obtained in a moderate yield (36%). However, cyclization with isophthaloyl dichloride bearing shorter alkyl chains failed to give macrocycle **2b,** most likely because of the precipitation of the precursors before completing a full turn of the macrocyclic backbone-a solubility issue often encountered in macrocyclization [56]. To improve the yield of cyclized products, H-bonded macrocycles were prepared by an alternative condensation protocol involving the [4 + 1] cyclization of a tetrameric diacid dichloride **13′** and azobenzene diamine **12** (Scheme 2). Compound **13** is derived from the hydrolysis of its corresponding ester, which is obtained by Bop-Cl-promoted condensation of isophthalic acid **5** and **12** (Scheme S1). A relatively high-yielding synthesis of macrocycles **2a**–**2d** with yields varying from 36% to 60% is achieved. The identities of these macrocycles were confirmed by ^1^H NMR, ^13^C NMR, and ESI-HRMS spectrometry, with the exception of **2b**, for which only ESI-HRMS data could be obtained due to solubility limitations (Figures S1–S6, S20–S23). The enhanced yields of these products indicate that using a larger H-bonded oligomeric precursor favors the formation of cyclized products. We ascribe it to the template effect of triethylamine hydrochloride on the macrocyclization [13,15].

**Figure 2:**
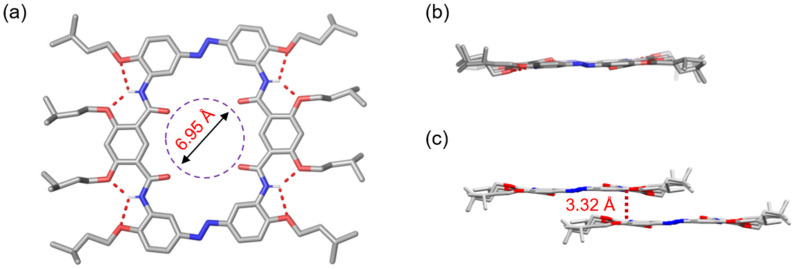
Single-crystal X-ray structure of macrocycle **2d** (color code: gray, carbon; blue, nitrogen; and red, oxygen) showing (**a**) top (red dashed lines indicate the presence of intramolecular hydrogen bonds) and (**b**) side views, and (**c**) the dimeric stacking of macrocycle **2d** in the solid state (the red dashed line indicates the π–π stacking interactions between two macrocycles). For clarity, only hydrogen atoms involved in hydrogen bonding are shown. Intramolecular H-bond lengths [Å] (angles [°]): N1–H1···O5 2.23 (107), N1–H1···O6 1.96 (138), N2–H2···O7 2.25 (106), N2–H2···O8 1.93 (138), N3–H3···O9 1.93 (138), N3–H3···O10 2.25 (138), N4–H4···O11 1.96 (138), and N4–H4···O12 2.23 (107).

Single crystals of macrocycle **2d**, which shares the same backbone as **2a** but bears different side chains, were obtained by slow evaporation from a 2:1 CHCl_3_/CH_3_OH solution. Macrocycle **2d** crystallizes in the triclinic *P*-1 space group with lattice parameters *a* = 17.6382(4) Å, *b* = 10.2115(2) Å, *c* = 23.2933(6) Å, α = 90°, β = 90.975°(2), and γ = 90° (Table S1). Single-crystal X-ray diffraction reveals a smaller hydrophilic cavity (6.95 Å) than that of macrocycle **1** (8.30 Å) (Figure 2a and Figure S25). The O···O distances between carbonyl oxygens within the same benzoyl unit (O1–O3) and between opposing benzoyl units (O1–O2) are 4.57 and 5.27 Å, respectively. The diagonal O···O distances (O1–O4 and O2–O3) are 6.48 and 7.42 Å, respectively, consistent with an approximately rectangular cavity defined by O1–O4 (Table S3). Within this cavity, two carbonyl oxygens point upward and two point downward, likely reflecting weak repulsive interactions among the carbonyl groups. Both azobenzene units adopt the *E* configuration and remain nearly planar (Figure 2b). In the solid state, adjacent macrocycles exhibit intermolecular π–π stacking with an interplanar distance of ~3.32 Å (Figure 2c). Notably, macrocycle **2c** is much more soluble than **2a**, **2b**, and **2d**, facilitating host–guest studies with various alkali metal ions.

Macrocycle **2** contains two azobenzene units cyclized through aromatic amide linkages. Because substituents on azobenzene strongly affect photoisomerization, we first characterized the switching behavior of **2c** by UV–vis absorption spectroscopy. A freshly prepared solution shows an intense π–π* band at λ_max_ = 372 nm and a weaker n–π* band at λ_max_ = 443 nm. Upon 365 nm irradiation, the π–π* band decreases markedly while the n–π* band increases slightly, consistent with *E*→*Z* photoisomerization. The photostationary state is reached within 1 min. Subsequent irradiation with blue light (450 nm) restores the *E*-isomer, demonstrating reversible switching (Figure S26). Under UV irradiation (365 nm; 50 μM in CHCl_3_/CH_3_CN = 2:1, *v*/*v*; 298 K), the photoisomerization rate constant is 5.34 × 10^−4^ ms^−1^ (Figure S27). The reversibility and fatigue resistance were then evaluated under the same conditions by repeated UV/blue-light cycling. The absorption changes remained essentially unchanged over at least six cycles, with no obvious photofatigue (Figure S28). These results indicate that the azobenzene units retain their intrinsic photoisomerization characteristics—comparable to those of conventional azo-macrocycles [57]—after incorporation into the hydrogen-bonded macrocyclic scaffold.

**Figure 3:**
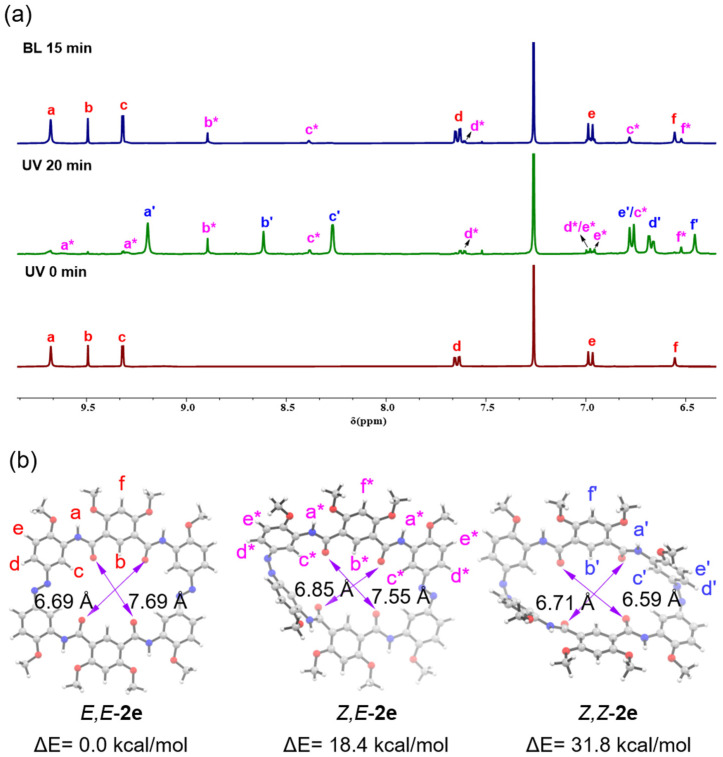
(**a**) ^1^H NMR spectra of macrocycle **2c** in CDCl_3_ (1 mM, 400 MHz, 298 K) showing photoisomerization. (**b**) DFT energy-minimized structures optimized at the B3LYP/6-31G level. Note: letters a–f, a*–f*, and a’–f’ indicate the aromatic and amide protons of the *E*,*E*-, *Z*,*E*-, and *Z*,*Z*-isomers, respectively.

To identify all photogenerated isomers, ^1^H NMR spectra were recorded in CDCl_3_/CD_3_CN (*v*/*v*, 2:1). The freshly prepared solution of **2c** showed six well-resolved resonances in the aromatic and amide regions, indicating that *E*,*E*-**2c** is the exclusive isomer in the initial state (Figure S29a). Upon 365 nm irradiation, two new groups of signals appeared between 6.5 and 10 ppm. The dominant set is assigned to *Z*,*Z*-**2c**, consistent with the characteristic spectral changes in photoresponsive azo-macrocycles (Figure S29b). The weaker set—comprising four singlets together with several doublets and multiplets—reflects reduced symmetry of the macrocycle and is therefore attributed to the asymmetric *Z*,*E*-**2c** isomer. After 30 min of 365 nm irradiation, the system in CDCl_3_/CD_3_CN (*v*/*v*, 2:1) reached the photostationary state, at which integration of diagnostic resonances reveals a mixture of three isomers: *E*,*E*-**2c** (32%), *Z*,*E*-**2c** (16%), and *Z*,*Z*-**2c** (52%). In CDCl_3_ (Figure 3a), UV irradiation (365 nm, 20 min) drives the system predominantly to *Z,Z*-**2c**, with *Z,E*-**2c** present only as a minor component. Subsequent blue-light irradiation (450 nm for 15 min) converts >90% of the sample back to *E*,*E*-**2c**, leaving only weak residual signals from *Z*,*E*-**2c**. Moreover, solid-state photoisomerization of macrocycle **2** was not observed under 365 nm irradiation (Figure S30).

**Figure 4:**
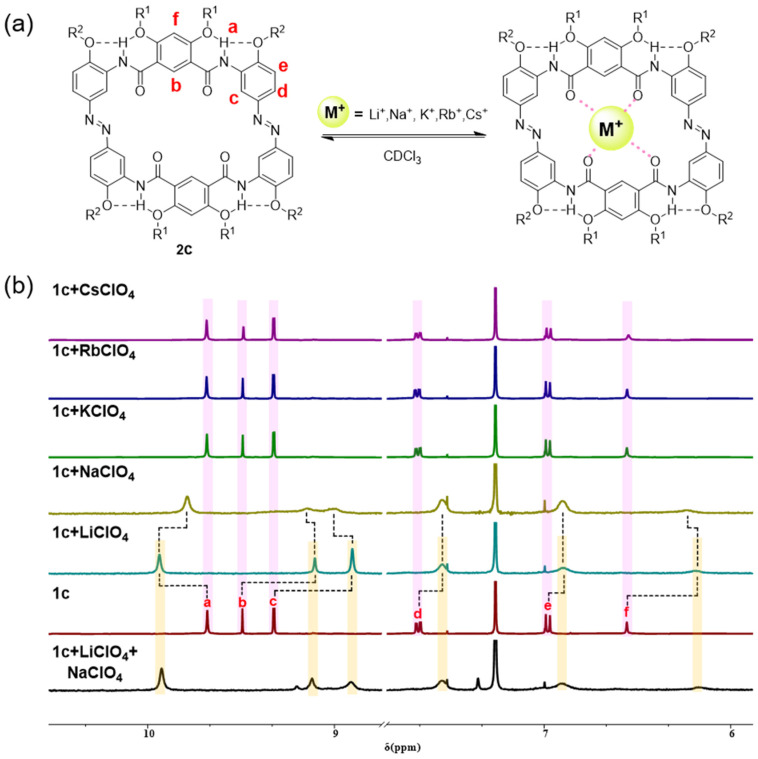
(**a**) Schematic illustration of host–guest interactions between azo-macrocycle **2c** and alkali-metal cations. (**b**) Partially stacked ^1^H NMR spectra (400 MHz, 298 K, CDCl_3_) of **2c** (1.0 mM) recorded in the absence and presence of 2.0 equiv. of Li^+^, Na^+^, K^+^, Rb^+^, and Cs^+^ (added as the corresponding ClO_4_^−^ salts). Note: letters a–f indicate the aromatic and amide protons of azo-macrocycle **2c**.

To verify our hypothesis that the new azo-macrocycle functions as a receptor for alkali metal cations (Li^+^, Na^+^, K^+^, Rb^+^, and Cs^+^ as their ClO_4_^−^ salts), we performed a screening study using **2c** in CDCl_3_ by ^1^H NMR spectroscopy. As shown in Figure 4b, adding excess Li^+^ or Na^+^ produced clear chemical-shift changes in aromatic resonances associated with the inward-facing binding site, whereas K^+^, Rb^+^, and Cs^+^ caused no appreciable spectral perturbation. The lack of detectable binding for K^+^, Rb^+^, and Cs^+^ is plausibly attributable to their larger ionic radii, which are incompatible with effective encapsulation in the receptor cavity. For Li^+^, the amide NH resonance (H^a^) shifted downfield by *Δ*δ = 0.26 ppm, while the interior aromatic protons (H^b^ and H^c^) shifted upfield by *Δ*δ = −0.39 and −0.42 ppm, respectively, relative to free **2c**. In contrast, Na^+^ induced a smaller downfield shift in H^a^ (*Δ*δ = 0.11 ppm) and upfield shifts in H^b^ and H^c^ (*Δ*δ = −0.35 and −0.32 ppm, respectively). These results indicate that **2c** engages in pronounced host–guest complexation selectively with Li^+^ and Na^+^ under these conditions. To enable direct comparison with previously reported azo-macrocycles and to minimize solubility-related differences arising from side-chain variation, we also prepared a reference macrocycle **1** bearing the same alkyl substituents as **2c** using the established route (Scheme S2) [19]. Consistent with **2c**, qualitative binding assays show that **1** exhibits discernible interactions only with Li^+^ and Na^+^ among the tested alkali metal cations (Figure S31).

To quantify the cation binding properties of macrocycles **1** and **2c**, ^1^H NMR titration experiments with LiClO_4_ and NaClO_4_ were performed in CDCl_3_ (Table 1). Upon addition of LiClO_4_ to **2c**, two distinct sets of resonances appeared and persisted until saturation at 1.0 equiv. of LiClO_4_. These signals correspond to free **2c** and the Li^+^-bound complex, indicating 1:1 complexation with slow exchange on the NMR timescale (Figure S32). In contrast, titration of **2c** with Na^+^, as well as titration of **1** with either Li^+^ or Na^+^, produced only single, continuously shifting resonances, consistent with fast exchange (Figures S33, S36 and S39). From the titration data, the association constant (*K*_a_) for **2c·**LiClO_4_ was estimated by a single-point method (see Supporting Information for details) to be >10^4^ M^−1^. This value is at least three orders of magnitude larger than that of receptor **1** toward LiClO_4_ (*K*_a_ < 5 M^−1^) determined under the same conditions. By comparison, both receptors exhibit only moderate affinity for Na^+^, with *K*_a_ values of (1.25 ± 0.27) × 10^3^ M^−1^ for **2c** and (6.61 ± 1.76) × 10^2^ M^−1^ for **1** (Figures S35, S38 and S41). Collectively, these results show that **2c** binds Li^+^ strongly and selectively over Na^+^, whereas **1** displays weak binding toward Li^+^ and Na^+^ with moderate selectivity for Na^+^.

**Figure 5:**
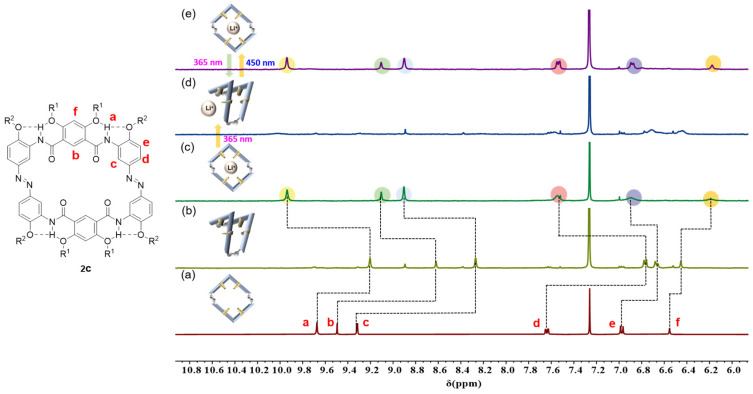
Partial ^1^H NMR spectra (400 MHz, 298 K, CDCl_3_) of **2c** (1.0 mM): (**a**) before photoirradiation at 365 nm; (**b**) after addition of 2.0 equiv. of LiClO_4_; (**c**) after photoirradiation at 365 nm; (**d**) after photoirradiation at 365 nm of the LiClO_4_-containing sample shown in (**b**); and (**e**) after subsequent photoirradiation at 450 nm of the sample shown in (**d**). Note: letters a–f indicate the aromatic and amide protons of azo-macrocycle **2c**.

Building on this distinct Li^+^ preference, **2c** was further employed to investigate its potential as an effective photoswitchable cation receptor capable of reversibly light-triggered binding of Li^+^. As shown in Figure 5, spectroscopic analysis of compound **2c** in CDCl_3_ at room temperature displays a single set of resonances (Figure 5a), indicating that it exists predominantly as the *E*,*E*-**2c** isomer. Irradiation of the solution at 365 nm for 30 min generated a new set of signals attributable to *Z*,*Z*-**2c**, demonstrating efficient *E*↔*Z* photoisomerization of the constituent azobenzene units (Figure 5b). When the pre-formed *E*,*E*-**2c** ⊃ LiClO_4_ complex was irradiated at 365 nm (Figure 5c), the ^1^H NMR signals became markedly broadened (Figure 5d). A subsequent ^1^H NMR titration of the irradiated macrocycle **2c** with LiClO_4_ (Figure S42) revealed a transition of Li^+^ binding from slow exchange to fast exchange binding regime on the NMR timescale during the UV-induced switching process. Notably, subsequent irradiation of the *Z*,*Z*-rich mixture with visible light (450 nm) for up to 20 min restored the *E*,*E*-**2c** isomer nearly quantitatively and simultaneously regenerated the *E*,*E*-**2c** ⊃ LiClO_4_ complex. The resulting spectrum is essentially identical to that of the pristine complex (Figure 5e vs. Figure 5c), evidencing reversibly light-triggered binding of Li^+^ driven by *Z*→*E* photoisomerization of macrocycle.

## 3. Materials and Methods

### 3.1. Materials and Purifications

Chemical reagents used for macrocycle synthesis were purchased from Energy Chemical Co., Ltd. (analytically pure, Shanghai, China) and Sigma-Aldrich (Shanghai, China) in their purest forms. All chemicals were purchased from commercial suppliers and used without further purification unless otherwise stated. Column chromatography was performed on silica gel (100–200 mesh or 300–400 mesh, Qingdao Haiyang Chemical Co., Ltd., Qingdao, China). Reaction progress was monitored by thin-layer chromatography (TLC). Solvents used for extraction and chromatography were of reagent grade.

### 3.2. Experimental Methods

Analytical NMR spectra were recorded on Bruker AVANCE AV II-400 MHz (Bruker, Karlsruhe, Germany) at a constant temperature of 298 K. Chemical shifts are reported in δ values in ppm using tetramethylsilane (TMS) or residual solvent as internal standard, and coupling constants (*J*) are denoted in Hz. Multiplicities are denoted as follows: s = singlet, d = doublet, t = triplet, dd = double doublet, and m = multiplet. Electrospray ionization high resolution mass (ESI-HRMS) data were collected by WATERS Q-TOF Premier (SHIMADZU, Kyoto, Japan). Ultraviolet–Visible Spectroscopy Experiments: Unless otherwise noted, the testing solvents were all HPLC-grade water. UV-vis spectra were measured by SHIMADZU UV-2600i (SHIMADZU, Kyoto, Japan). Single crystal X-ray data were measured on a Xcalibur E diffractometer (Oxford Diffraction Ltd., Abingdon, UK) with graphite monochromated Mo-Kα radiation (λ = 0.71073 Å). Data collection and structure refinement details can be found in the CIF files or obtained free of charge via https://www.ccdc.cam.ac.uk/ (accessed on 24 February 2026).

### 3.3. Synthesis of Macrocycles ***1*** and ***2***

Macrocycles **2a**–**2d** were synthesized following the procedure described in the Supporting Information (Scheme S1). Macrocycle **1** was synthesized according to previous references [19] (Scheme S2).

### 3.4. DFT Calculations

DFT calculations for the geometrical optimizations were performed in the Gaussian 09 program package (Gaussian, Inc., Wallingford, CT, USA). Geometry optimizations of all minima and intermediates were carried out using the Becke three-parameter hybrid functional (B3LYP) [58] and the 6-31G(d)/6-311G(d,p) basis set for C, H, N, and O atoms. All substituents (R) on the periphery of macrocycle **2** are replaced by methyl groups to give compound **2e** for simplicity.

## 4. Conclusions

In summary, we developed a new class of photoresponsive, hydrogen-bonded azo-macrocycles (**2**) by integrating azobenzene photoswitches into macrocyclic cation receptors. Rewiring the connectivity between the amide linkages and the azobenzene units redistributes electron density and preorganizes the carbonyl donors to generate a more confined binding cavity, thereby enhancing metal-ion affinity. Notably, macrocycle **2** displays pronounced selectivity for Li^+^ among the alkali metal ions and forms a Li^+^ complex that exchanges slowly on the ^1^H NMR timescale. Moreover, Li^+^ binding can be reversibly regulated by alternating UV and visible-light irradiation. Collectively, these results establish connectivity-reconfigured hydrogen-bonded azo-macrocycles as a versatile platform for photoswitchable ion recognition and suggest a general design strategy for energy-efficient, selective separations.

## Figures and Tables

**Table 1:** Association constants (*K*_a_, M^−1^) alkali metal binding with hydrogen-bonded azo-macrocycles **1** and **2** in CDCl_3_ at 298 K.

Complex *^a^*	NMR H–G Exchange	*K*_a_ (M^−1^) *^b^*
**2c** ⊃ LiClO_4_	Slow	>10^4^ *^c^*
**1** ⊃ LiClO_4_	Fast	<5 *^d^*
**2c** ⊃ NaClO_4_	Fast	(1.25 ± 0.27) × 10^3^ *^d^*
**1** ⊃ NaClO_4_	Fast	(6.61 ± 1.76) × 10^2^ *^d^*

*^a^* Job plot analysis indicates that all complexes exhibit a 1:1 stoichiometry (Figures S34, S37 and S40). *^b^* Association constants (*K*_a_) were determined by ^1^H NMR titration experiments; *^c^* For the slow exchange case, *K*_a_ was estimated using the single-point method (see the Supporting Information for details). *^d^ K*_a_ values were fitted using BindFit v0.5 available from https://app.supramolecular.org/bindfit (accessed on 9 December 2025).

## Data Availability

Data are contained within the article and Supplementary Materials.

## Supplementary Materials

The following supporting information can be downloaded at https://www.mdpi.com/article/10.3390/molecules31071086/s1. Scheme S1: Synthesis of azo-macrocycle **2**; Scheme S2: Synthetic route of azo-macrocycle **1**; Figures S1–S7: ^1^H NMR and ^13^C NMR spectrum of **1**, **2a**, **2c** and **2d**; Figures S8–S19: ^1^H NMR and ^13^C NMR spectrum of key intermediates; Figures S20–S23: ESI-HRMS spectrum of **2a**, **2b**, **2c** and **2d**; Figures S24 and S25: Crystal structure of macrocycle **2d**; Table S1: Crystallographic data of **2d**; Table S2: Intramolecular hydrogen bond parameters of **2d**; Table S3: Selected interatomic distances of **2d**; Figure S26: UV–vis absorption spectra of **2c**; Figure S27: Photoisomerization rate of **2c**; Figure S28: Absorbance intensity changes of **2c**; Figure S29: Stacked partial ^1^H NMR spectra of **2c**; Figure S30. IR spectra of macrocycle **2** in the solid state before and after irradiation with 365 nm light; Figure S31: Stacked partial ^1^H NMR spectra of **1**; Figures S32–S42: ^1^H NMR titration of **1** (**2c**) with LiClO_4_ (NaClO_4_); Figure S43: DFT-optimized structures of **2e**; Figure S44: Structures, relative energies of the three stereoisomers of **2e**. References [19,59,60,61] are cited in the Supplementary Materials.
