# Supplementary material for: Connectivity-Rewired Construction of Hydrogen-Bonded Azo-Macrocycles Enables Photoswitchable Recognition of Lithium Ions
Source: Molecules. 2026 Mar 26;31(7):1086. doi: 10.3390/molecules31071086 (PMC13075020; doi:10.3390/molecules31071086)
Supplement: Supplementary file 1 [file molecules-31-01086-s001.zip › molecules-4203325-supplementary.pdf]

Supporting Information

Connectivity-Rewired Construction of Hydrogen-Bonded Azo-Macrocycles Enables Photoswitchable Recognition of Lithium Ions

Chengyu Tan †, Kuirong Fu †, Zhiyao Yang, Song Qin, Yimin Cai, Wen Feng, Xiaowei Li * and Lihua Yuan *

College of Chemistry, Key Laboratory of Radiation Physics and Technology of Ministry of Education, Institute of Nuclear Science and Technology, Sichuan University, Chengdu 610064, China;
chengyutan2001@163.com (C.T.); frederrean@163.com (K.F.); yangzhiyaohaha@sina.com (Z.Y.);
qinsong@scu.edu.cn (S.Q.); ymcai@scu.edu.cn (Y.C.); wfeng9510@scu.edu.cn (W.F.)

* Correspondence: lixw@scu.edu.cn (X.L.); lhyuan@scu.edu.cn (L.Y.)

† These authors contributed equally to this work.

Table of Contents

1. Materials and methods 3

2. Synthesis and characterization 4

2.1 Synthesis of macrocycle **2** 5

2.2 Synthesis of macrocycle **1** 19

2.3 Characterizations 20

2.3.1 NMR spectra 20

2.3.2 Mass spectra 29

3. X-ray crystal structures 31

4. Photoisomerization studies 34

5. Recognition of alkali metal salts 37

6. DFT calculations 44

References 46

1. Materials and methods

All chemicals were purchased from commercial suppliers and used as received unless otherwise stated. Reactions were performed in oven-dried glassware under an atmosphere of air or nitrogen, as appropriate, and stirred with Teflon-coated magnetic stir bars. Solvents were dried and distilled according to standard procedures. Reaction progress was monitored by thin-layer chromatography (TLC). Flash column chromatography was carried out on silica gel (100–200 mesh or 300–400 mesh). Solvents used for extraction and chromatography were of reagent grade. Deuterated NMR solvents were purchased from Cambridge Isotope Laboratories (CIL).

NMR spectra were recorded on Bruker AVANCE spectrometers (400 or 600 MHz) at 298 K unless otherwise noted. Chemical shifts (δ) are reported in ppm relative to tetramethylsilane (TMS) or residual solvent signals as internal references, and coupling constants (*J*) are reported in Hz. Signal multiplicities are abbreviated as: s (singlet), d (doublet), t (triplet), dd (doublet of doublets), and m (multiplet). High-resolution mass spectra (ESI-HRMS) were acquired on a WATERS Q-TOF Premier instrument. UV–vis absorption spectra were recorded on a SHIMADZU UV-2600i spectrophotometer using HPLC-grade solvents (unless otherwise specified). Single-crystal X-ray diffraction data were collected on an Xcalibur E diffractometer with graphite-monochromated Mo Kα radiation (λ = 0.71073 Å). Crystallographic data and refinement details are provided in the corresponding CIF files, which are available from the Cambridge Crystallographic Data Centre (CCDC) via the link: https://www.ccdc.cam.ac.uk/structures/.

2. Synthesis and characterization

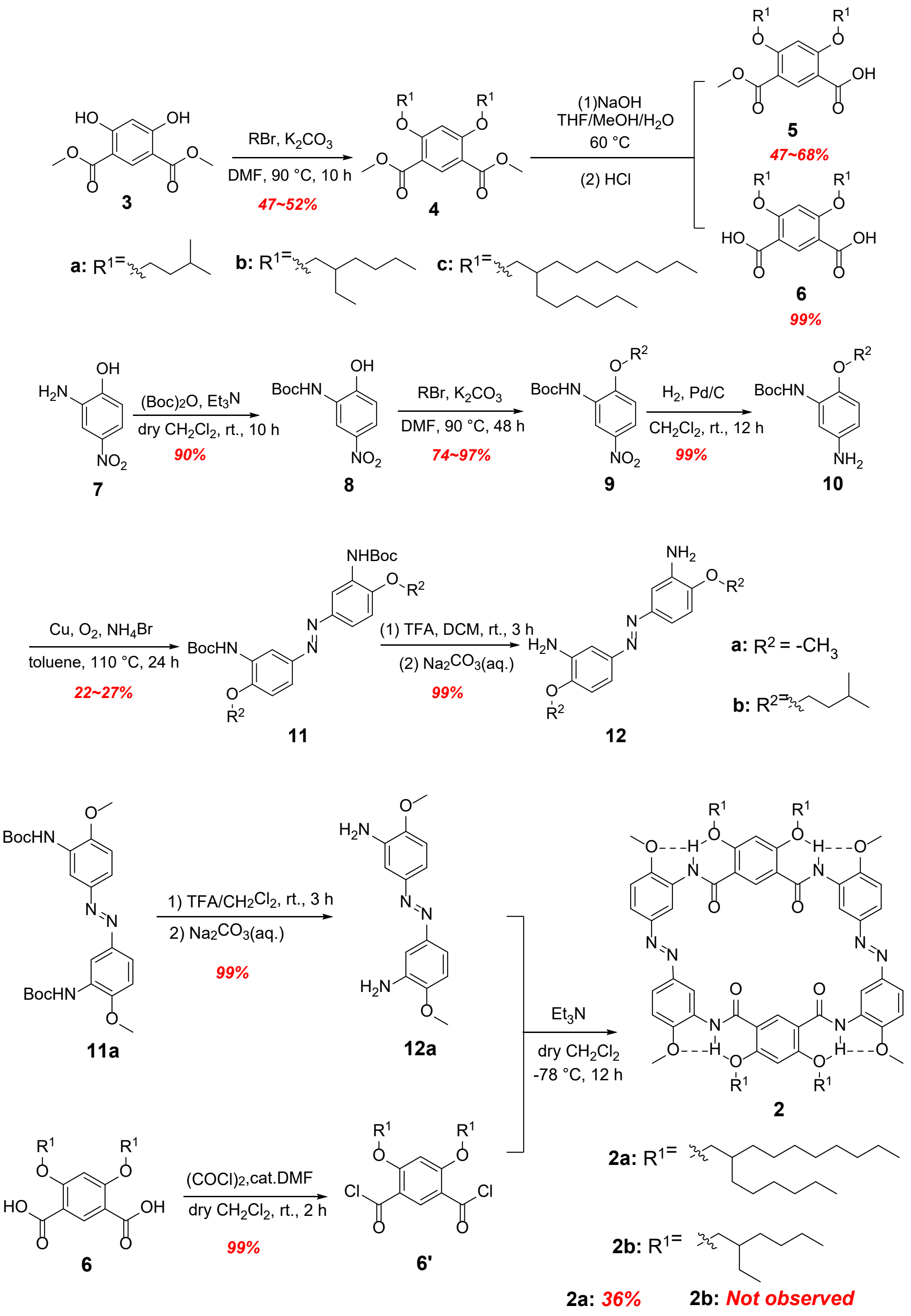

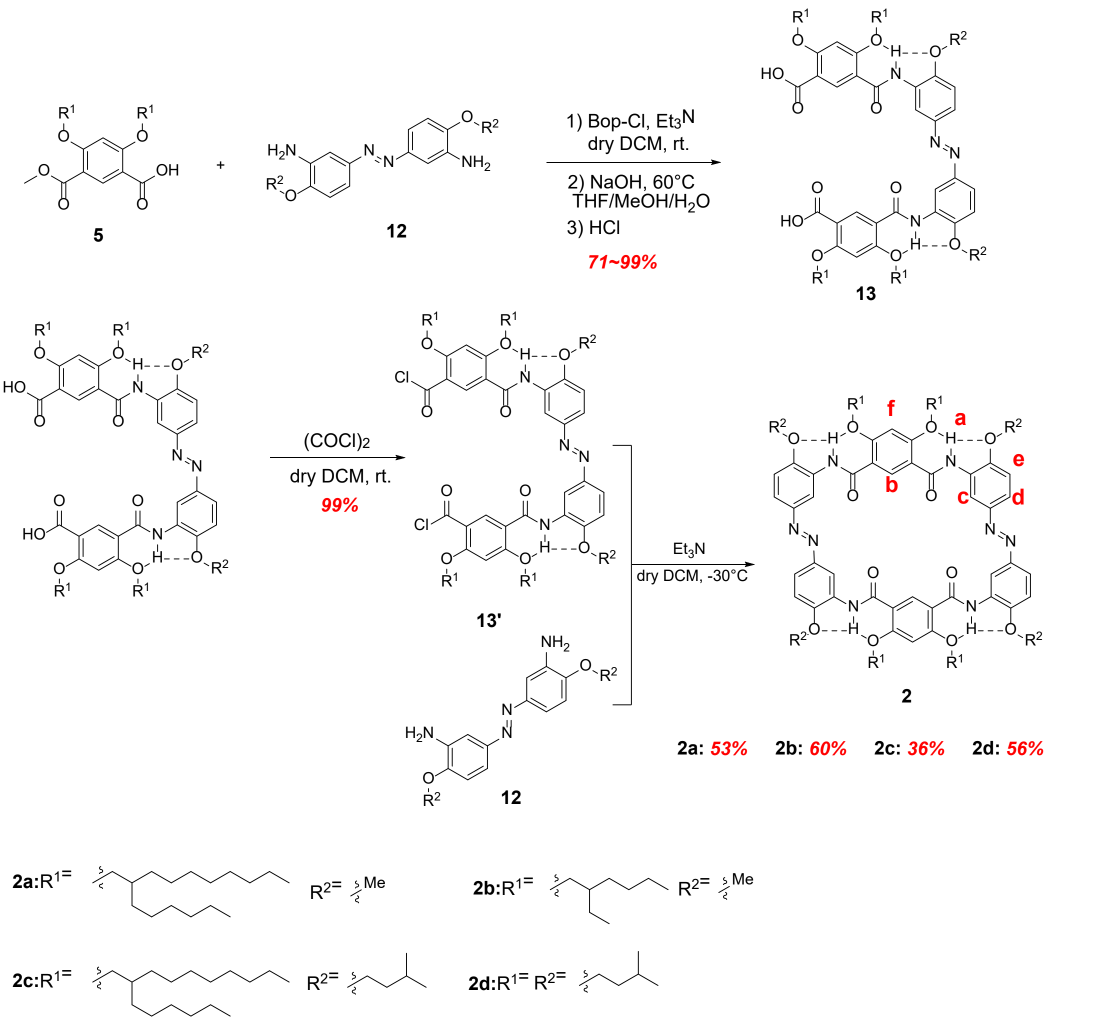

Scheme S1. Synthesis of azo-macrocycle **2**.

2.1 Synthesis of macrocycle 2

Compounds **4-6** and **8-10** were synthesized according to similar procedures in the literature[1-3].

Synthesis of monomer **4a**

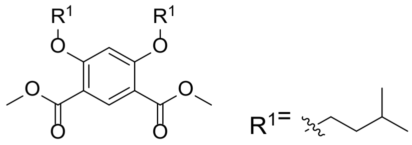

A mixture of **3** (10.0 g, 44.2 mmol) and K_2CO_3 (18.3 g,132.6 mmol) in 200 mL *N*,*N'*-dimethylformamide (DMF) was stirred at 65 °C for 2 h, and then 1-bromo-3-methylbutane

(92.8 mmol, 2.1 eq.) was added to the mixture, followed by increasing the temperature to 110°C for another 10 h. The solid residue was removed by filtration, and the solvent was evaporated under high vacuum. The residue was dissolved in ethyl acetate and washed with water (3 × 50 mL). The crude material was purified using column chromatography (eluent: hexane/ethyl acetate = 30/1, v/v) to afford compound **4a** as a yellow oil (8.43 g, 52%). ^{1}H NMR (400 MHz, $CDCl_3$): δ 8.46 (s, 1H), 6.45 (s, 1H), 4.10 (t, *J* = 6.6 Hz, 4H), 3.85 (s, 6H), 1.95-1.88 (m, 2H), 1.77 (q, *J* = 6.6 Hz, 4H), 0.98 (d, *J* = 6.6 Hz, 12H).

Synthesis of monomer **4b**

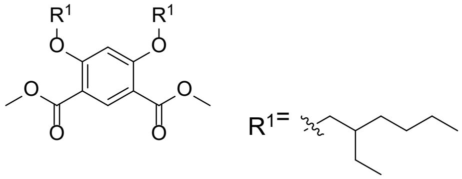

Following a procedure similar to that described for compound **4a** using R^{1}Br and compound **3** as the starting material, **4b** was obtained as an oil in 50% yield.

Synthesis of monomer **4c**

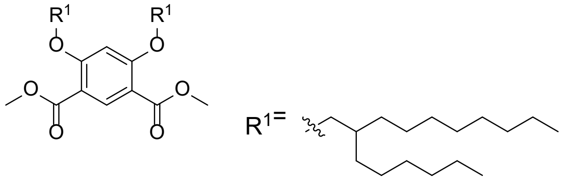

Following a procedure similar to that described for compound **4a** using R^{1}Br and compound **3** as the starting material, **4c** was obtained as an oil in 47% yield.

Synthesis of monomer **5a**

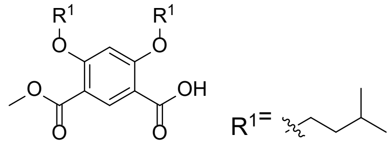

To a solution of compound **4a** (5.00 g, 13.6 mmol, 1.0 eq.) in anhydrous methanol (100 mL) was added a saturated aqueous solution of NaOH (1.2 eq.). The reaction mixture was stirred under reflux for 3 h. The reaction was monitored by TLC and was quenched when most of the starting material had been consumed, but before significant formation of the bis-hydrolyzed by-product occurred. Diluted HCl aq. was added to the solution until pH=2. The solvent was removed under reduced pressure, and the residue was dissolved in CH_2Cl_2 and washed with water (3 × 50 mL). The crude material was purified using column chromatography (eluent: CH_2Cl_2) to afford compound **5a** as a yellow oil (2.55 g, 53%). ^1H NMR (400 MHz, $CDCl_3$): δ 10.45 (s, 1H), 8.69 (s, 1H), 6.51 (s, 1H), 4.30 (t, *J* = 6.5 Hz, 2H), 4.13 (t, *J* = 6.5 Hz, 2H), 3.86 (s, 3H), 1.97-1.74 (m, 6H), 1.04-0.96 (m, 12H).

Synthesis of monomer **5b**

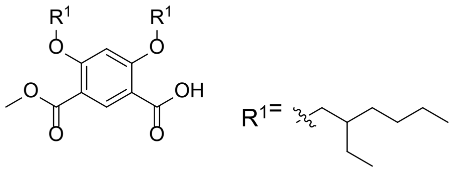

Following a procedure similar to that described for compound **5a** using compound **4b** as the starting material, **5b** was obtained as an oil in 68% yield.

Synthesis of monomer **5c**

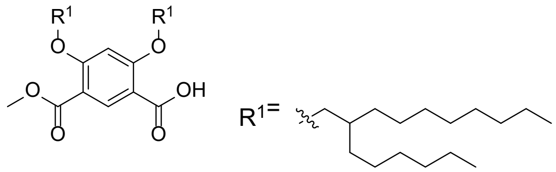

Following a procedure similar to that described for compound **5a** using compound **4c** as the starting material, **5c** was obtained as an oil in 47% yield. ^1H NMR (400 MHz, $CDCl_3$):

δ 10.47 (s, 1H), 8.70 (s, 1H), 6.48 (s, 1H), 4.16 (d, J = 5.2 Hz, 2H), 3.97 (d, J = 5.2 Hz, 2H), 3.86 (s, 3H), 1.93-1.81 (m, 2H), 1.57-1.18 (m, 53H), 0.95-0.82 (m, 12H).

Synthesis of monomer **6a**

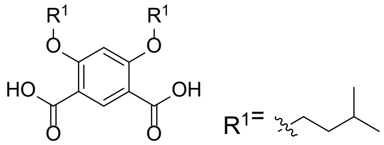

To a solution of compound **4a** (5.00 g, 13.6 mmol, 1.0 eq.) in anhydrous methanol (100 mL) was added a saturated aqueous solution of NaOH (3.0 eq.). The reaction mixture was stirred under reflux at 90 °C for 6 h. Diluted HCl aq. was added to the solution until pH=2, resulting in the formation of a substantial amount of white solid. The resulting white precipitate was°C ollected by filtration, dried, and isolated as the product (4.55 g, 99%). ^{1}H NMR (400 MHz, DMSO-d_6): δ 12.33 (s, 2H), 8.17 (s, 1H), 6.70 (s, 1H), 4.15 (t, J = 6.6 Hz, 4H), 1.88-1.81 (m, 2H), 1.63 (q, J = 6.6 Hz, 4H), 0.93 (d, J = 6.6 Hz, 12H).

Synthesis of monomer **6b**

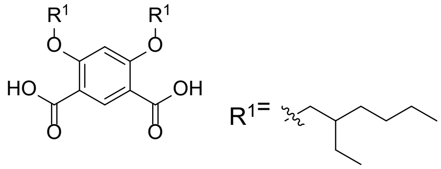

Following a procedure similar to that described for compound **6a** using compound **4b** as the starting material, **6b** was obtained as a white solid in 99% yield.

Synthesis of monomer **6c**

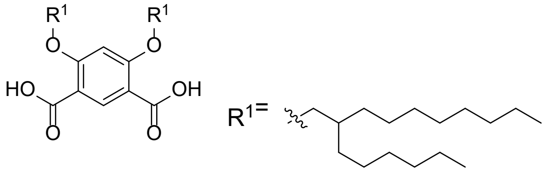

Following a procedure similar to that described for compound **6a** using compound **4c** as the starting material, **6c** was obtained as a white solid in 99% yield. ^{1}H NMR (400 MHz, $CDCl_3$): δ 10.39 (s, 2H), 8.95 (s, 1H), 6.55 (s, 1H), 4.15 (d, *J* = 5.2 Hz, 5H), 1.96-1.87 (m, 2H), 1.51-1.43 (m, 10H), 1.41-1.19 (m, 48H), 0.91-0.85 (m, 12H).

Synthesis of monomer **8**

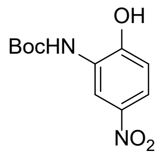

A mixture of 4-amino-2-nitrophenol (6.00 g, 43.2 mmol) and di-tert-butyl dicarbonate (12.0 g, 55.0 mmol) in tetrahydrofuran (60 mL) was heated at 55°C for 10 h. After the reaction mixture was cooled to room temperature, the solvent was removed under reduced pressure. The residue was treated with cyclohexane (100 mL), which induced the precipitation of a yellow solid. The solid was collected by filtration to afford compound **8** as a yellow solid (8.90 g, 90%). ^{1}H NMR (400 MHz, $CDCl_3$): δ 9.29 (s, 1H), 8.15 (d, *J* = 2.6 Hz, 1H), 7.95 (dd, *J* = 8.9, 2.6 Hz, 1H), 7.01 (d, *J* = 8.9 Hz, 1H), 6.77 (s, 1H), 1.55 (s, 9H).

Synthesis of monomer **9a**

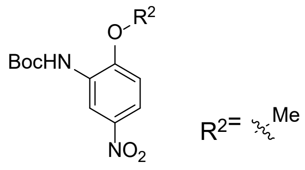

A mixture of 2-methoxy-5-nitroaniline (16.8 g, 0.1 mol) and di-tert-butyl dicarbonate (43.7 g, 0.2 mol) in ethanol (170 mL) was stirred at room temperature for 2 days. A significant amount of bright yellow solid precipitated during the reaction. The solid was collected by filtration, washed with a small amount of ethanol, and dried to afford compound **9a** as a yellow solid (19.9 g, 74%). ^{1}H NMR (400 MHz, $CDCl_3$): δ 9.01 (s, 1H), 7.92 (dd, *J* = 9.0, 2.8 Hz, 1H), 7.13 (s, 1H), 6.89 (d, *J* = 9.0 Hz, 1H), 3.99 (s, 3H), 1.55 (s, 9H).

Synthesis of monomer **9b**

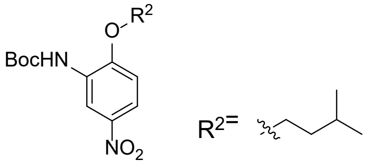

A mixture of **8** (5.00 g, 19.7 mmol) and K_2CO_3 (13.6 g, 98.3 mmol) in 50 mL N,N'-dimethylformamide (DMF) was stirred at 65 °C for 2 h, and then 1-bromo-3-methylbutane (59.0 mmol, 3.0 eq.) was added to the mixture, followed by increasing the temperature to 80 °C for another 6 h. The solid residue was removed by filtration, and the solvent was evaporated under high vacuum. The residue was dissolved in ethyl acetate and washed with water (3 × 50 mL). The crude material was purified using column chromatography (eluent: hexane/ CH_2Cl_2 = 3/1, v/v) to afford **9b** as a yellow oil (6.20 g, 97%). ^{1}H NMR (400 MHz, $CDCl_3$): δ 9.00 (s, 1H), 7.90 (dd, J = 9.0, 2.8 Hz, 1H), 7.06 (s, 1H), 6.88 (d, J = 9.0 Hz, 1H), 4.16 (t, J = 6.5 Hz, 2H), 1.86-1.76 (m, 3H), 1.56 (s, 9H),1.01 (d, J = 6.5 Hz, 6H).

Synthesis of monomer **10**

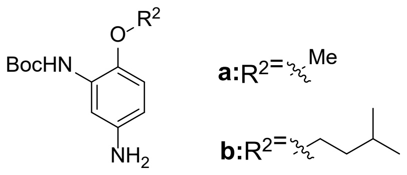

General procedure: The compound **9** (a or b) was dissolved in CH_2Cl_2 with Pd-C (10% weight), and the solution was stirred overnight under a H_2 atmosphere. Solid catalyst and solvent were removed by filtration, followed by evaporation under reduced pressure. The intermediate amine **10** was used for the next step without further purification.

Synthesis of dimer **11a**

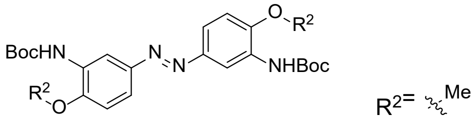

A mixture of compound **10a** (500 mg, 2.1 mmol), CuBr (301 mg, 2.1 mmol), and pyridine (498 mg, 6.3 mmol) in toluene (50 mL) was stirred at 60 °C under an O_2 atmosphere for 24 h. The crude material was purified by column chromatography (eluent: CH_2Cl_2/hexane = 3/1, v/v) to afford compound **11a** as an orange solid (110 mg, 22%). ^{1}H NMR (400 MHz, $CDCl_3$): δ 8.64 (s, 2H), 7.63 (dd, *J* = 8.7, 2.3 Hz, 2H), 7.10 (s, 2H), 6.95 (d, *J* = 8.7 Hz, 2H), 3.95 (s, 6H), 1.55 (s, 18H). ^{13}C NMR (100 MHz, $CDCl_3$): δ 154.4, 149.6, 148.2, 129.9, 120.9, 113.0, 110.1, 57.4, 30.0.

Synthesis of dimer **11b**

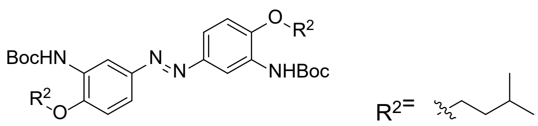

Following a procedure similar to that described for compound **11a** using compound **10b** as the starting material, **11b** was obtained as an orange solid in 27% yield. ^{1}H NMR (400 MHz, $CDCl_3$): δ 8.63 (s, 2H), 7.60 (dd, *J* = 8.7, 2.3 Hz, 2H), 7.06 (s, 2H), 6.94 (d, *J* = 8.7 Hz, 2H), 4.13 (t, *J* = 6.6 Hz, 4H), 1.89-1.74 (m, 6H), 1.56 (s, 19H), 1.01 (d, *J* = 6.6 Hz, 12H). ^{13}C NMR (100 MHz, $CDCl_3$): δ 152.7, 149.0, 147.2, 130.0, 120.8, 110.7, 110.5, 68.4, 37.9, 29.3, 25.4, 22.8.

Synthesis of dimer **12**

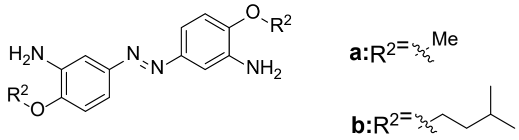

General procedure: A solution of protected tetramer **11** and excess trifluoroacetic acid (TFA) in 20 mL of CH_2Cl_2 was stirred at room temperature for 3 h. The solvent and excess TFA were removed under vacuum. The residue was washed with concentrated Na_2CO_3 aq. (3 × 50 mL). The organic phase was collected and dried over anhydrous Na_2SO_4; after filtration and evaporation, the dimer amine **12** was obtained and used for the next step without further purification.

Synthesis of monomer **6’**

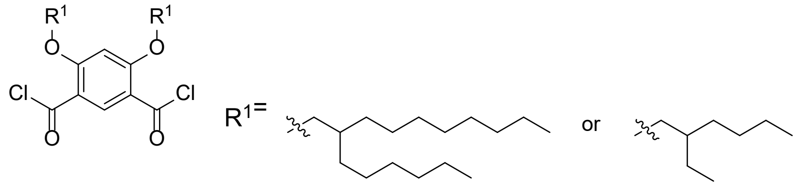

General procedure: A solution of compound **6** (1 eq.), oxalyl chloride (3 eq.), and 10 μL of dry DMF as initiator in dry CH_2Cl_2 was stirred for 2 h. The solvent and excess oxalyl chloride were removed under reduced pressure to afford the carbonyl chloride **6’**, which was used for the next step without further purification.

Synthesis of macrocycle **2a** via a [1+1] one-pot approach

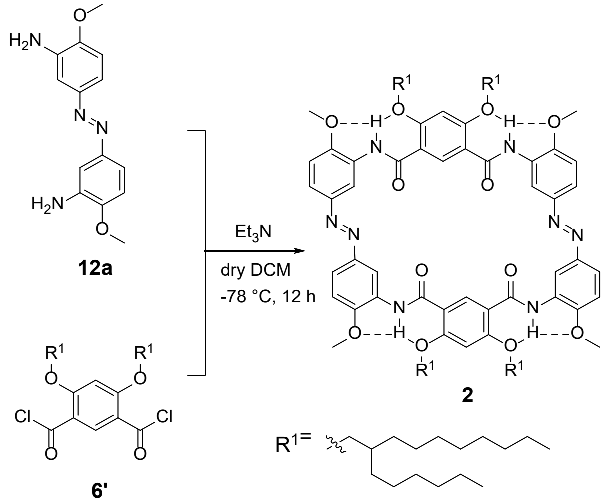

Compound **6’** (200 mg, 0.31 mmol) in CH_2Cl_2 (25 mL) was added dropwise to the solution of **12a** (84.2 mg, 0.31 mmol) and Et_3N (0.26 mL, 1.85 mmol) in CH_2Cl_2 (120 mL) at -78 °C. The solution was stirred under N_2 for 12 h. The organic layer was washed with water (3 × 50 mL) and dried over anhydrous Na_2SO_4. The crude material was purified using column chromatography (eluent: CH_2Cl_2/MeOH = 25/1, v/v) to afford compound **2a** as a yellow solid (94 mg, 34%). ^{1}H NMR (400 MHz, $CDCl_3$): δ 9.86 (s, 4H), 9.41 (s, 2H), 9.33 (d, *J* = 2.4 Hz, 4H), 7.69 (dd, *J* = 8.7, 2.4 Hz, 4H), 7.04 (d, *J* = 8.7 Hz, 4H), 6.58 (s, 2H), 4.18 (d, *J* = 5.9 Hz, 8H), 4.01 (s, 12H), 2.18-2.06 (m, 5H), 1.62-1.14 (m, 109H), 0.88-0.83 (m, 24H). ^{13}C NMR (100 MHz, $CDCl_3$/DMSO-*d*$_6$, 5:1, v/v): δ 181.5, 168.3, 162.5, 160.4, 150.6, 146.8, 144.2, 133.8, 129.0, 115.6, 73.3, 55.8, 40.5, 40.3, 40.1, 37.6, 31.5, 31.4, 30.7, 29.8, 29.4,

29.2, 29.0, 26.2, 22.3, 13.89, 13.86. ESI-HRMS m/z: $[C_{108}H_{166}N_8O_{12}+2H]^{2+}$ 884.1324;884.1306 was found.

Synthesis of tetramer **13a**

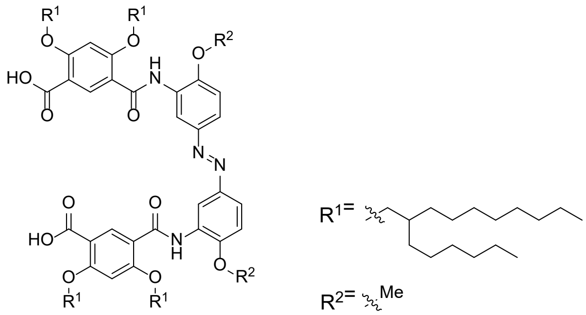

A solution of **5c** (2.1 eq.), Et_3N (4.0 eq.), and **12a** (1.0 eq.) in dry CH_2Cl_2 was stirred at r. t. for 12 h. After completion of the esterification, the solvent was removed under reduced pressure. The residue was dissolved in a mixture of methanol (80 mL) and tetrahydrofuran (80 mL). An aqueous solution of NaOH (380 mg, 9.6 mmol, 6.0 eq.) was added, and the reaction mixture was stirred under reflux at 80 °C for 12 h. The reaction progress was monitored by TLC, which indicated complete consumption of the starting material. Diluted HCl aq. was added to the solution until pH= 2, resulting in the formation of a substantial amount of yellow solid. The precipitate was collected by filtration, washed with water, and dried to afford the crude product. The crude material was purified using column chromatography (eluent: CH_2Cl_2/MeOH = 100/1, v/v) to afford compound **13a** as a yellow solid (2.10 g, 86%). ^{1}H NMR (400 MHz, $CDCl_3$): δ 10.26 (s, 2H), 9.80 (s, 2H), 9.14 (d, J = 2.3 Hz, 2H), 9.11 (s, 2H), 7.76 (dd, J = 8.7, 2.3 Hz, 2H), 7.02 (d, J = 8.7 Hz, 2H), 6.58 (s, 2H), 4.18-4.13 (m, 8H), 3.97 (s, 6H), 2.08-1.99 (m, 2H), 1.95-1.87 (m, 2H), 1.62-1.12 (m, 97H), 0.97-0.74 (m, 24H). ^{13}C NMR (100 MHz, $CDCl_3$): δ 164.3, 161.7, 161.3, 150.4, 147.3, 139.5, 128.4, 121.5, 117.1, 113.5, 111.1, 109.8, 96.7, 73.7, 73.4, 56.1, 38.0, 37.7, 32.00, 31.98, 31.9, 30.1, 30.0, 29.8, 29.7, 29.44, 29.43, 27.0, 26.9, 26.80, 26.77, 22.81, 22.78, 22.75, 14.23, 14.21, 14.19.

Synthesis of tetramer **13b**

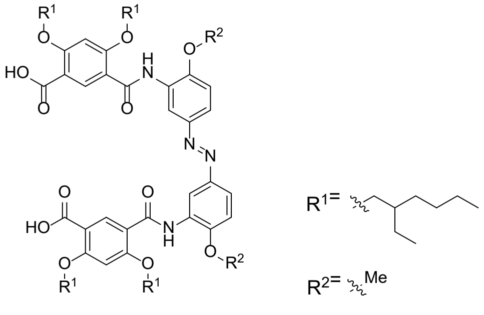

Following a procedure similar to that described for compound **13a** using compound **5b** and **12a** as the starting material, **13b** was obtained as an orange solid in 71% yield. ^{1}H NMR (400 MHz, $CDCl_3/CD_3OD$, 9:1, v/v): δ 9.96 (s, 1H), 9.07 (s, 2H), 8.91 (s, 2H), 7.76 (dd, *J* = 8.8, 2.3 Hz, 2H), 7.08 (d, *J* = 8.8 Hz, 2H), 6.60 (s, 2H), 4.23-4.05 (m, 8H), 4.00 (s, 6H), 2.07-1.94 (m, 2H), 1.89-1.82 (m, 2H), 1.67-1.17 (m, 32H), 1.05-0.72 (m, 24H). ^{13}C NMR (100 MHz, $CDCl_3/CD_3OD$, 9:1, v/v): δ 166.2, 162.5, 162.49, 161.5, 150.5, 147.0, 138.1, 128.0, 121.2, 115.1, 113.8, 112.6, 109.9, 96.8, 72.9, 72.2, 55.9, 39.2, 38.8, 30.7, 30.1, 29.0, 28.9, 28.3, 23.7, 23.5, 22.9, 22.8, 13.9, 13.8, 10.9, 10.6.

Synthesis of tetramer **13c**

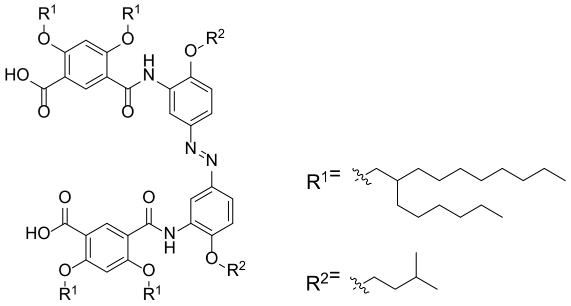

Following a procedure similar to that described for compound **13a** using compound **5c** and **12b** as the starting material, **13c** was obtained as an orange solid in 99% yield. ^{1}H NMR (400 MHz, $CDCl_3$): δ 10.25 (s, 2H), 9.62 (s, 2H), 9.11-9.03 (m, 3H), 7.76 (dd, *J* = 8.8, 2.4 Hz, 2H), 7.02 (d, *J* = 8.8 Hz, 2H), 6.58 (s, 2H), 4.24-4.09 (m, 10H), 2.06-1.88 (m, 4H), 1.79-1.73 (m, 6H), 1.53-1.11 (m, 92H), 0.98 (d, *J* = 6.2 Hz, 10H), 0.94-0.77 (m, 24H). ^{13}C NMR (100 MHz, $CDCl_3$): δ 164.2, 161.9, 161.7, 161.2, 150.4, 147.2, 139.8, 128.4, 122.5, 118.2, 115.2, 112.0, 111.0, 97.0, 73.8, 73.3, 67.9, 38.11, 38.06, 37.7, 32.00, 31.98, 31.91,

31.90, 31.44, 31.35, 30.1, 30.0, 29.8, 29.69, 29.67, 29.65, 29.43, 29.41, 27.01, 26.97, 26.9, 25.4, 22.82, 22.78, 22.7, 14.24, 14.21, 14.19.

Synthesis of tetramer **13d**

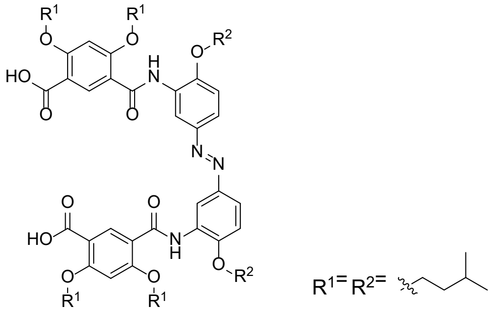

Following a procedure similar to that described for compound **13a** using compound **5a** and **12b** as the starting material, **13d** was obtained as an orange solid in 97% yield. ^{1}H NMR (400 MHz, $CDCl_3$/CD_3OD, 9:1, v/v): δ 9.95 (s, 1H), 9.04 (s, 2H), 8.88 (s, 2H), 7.74 (dd, *J* = 8.8, 1.9 Hz, 2H), 7.08 (d, *J* = 8.8 Hz, 2H), 6.62 (s, 2H), 4.36 (t, *J* = 6.9 Hz, 4H), 4.38-4.23 (m, 8H), 1.94-1.77(m, 18H), 1.03-0.97 (m, 36H). ^{13}C NMR (100 MHz, $CDCl_3$/CD_3OD, 9:1, v/v): δ 166.4, 162.5, 161.2, 150.7, 147.5, 137.9, 128.1, 121.6, 115.4, 114.1, 112.6, 111.5, 97.1, 68.7, 68.3, 67.7, 37.7, 37.4, 37.3, 25.1, 24.9, 22.5, 22.4, 22.3.

Synthesis of tetramer **13'**

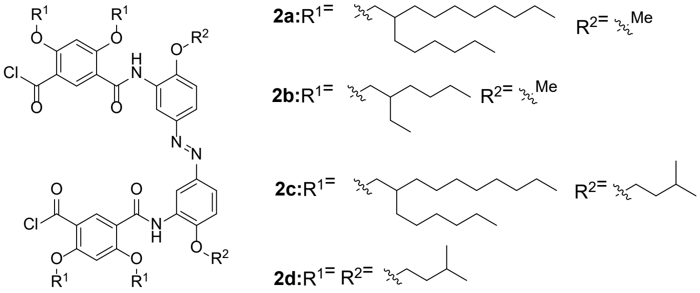

General procedure: A solution of compound **13** (1 eq.), oxalyl chloride (3 eq.), and 10 μL of dry DMF as initiator in dry CH_2Cl_2 was stirred for 0.5 h. The solvent and excess oxalyl chloride were removed under reduced pressure to afford the carbonyl chloride **13'**, which was used for the next step without further purification.

Synthesis of macrocycle **2** via a [4+1] coupling approach

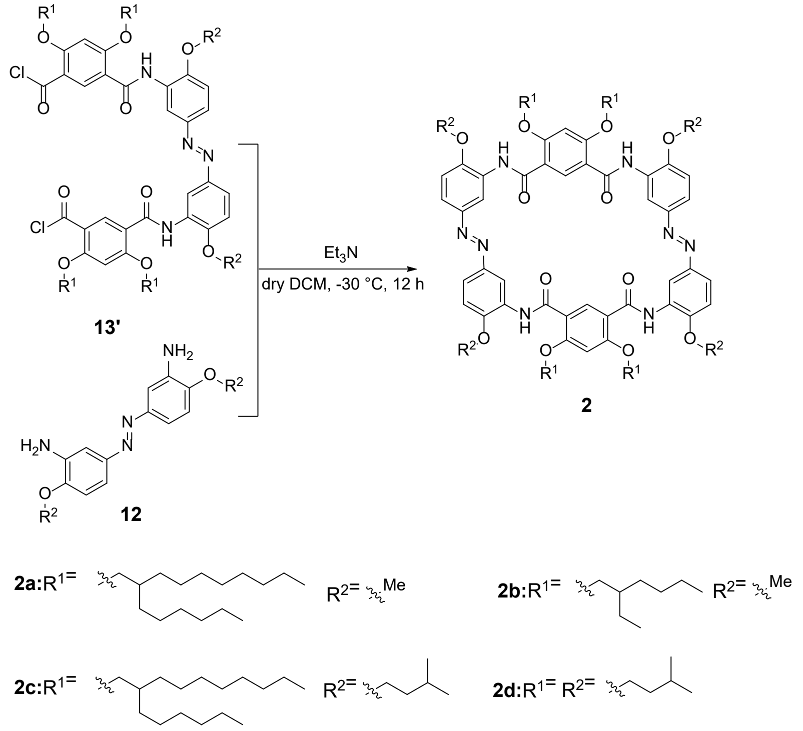

Synthesis of macrocycle **2a**

Compound **13'a** (300 mg, 0.2 mmol) in CH_2Cl_2 (30 mL) was added dropwise to the solution of **12a** (54.0 mg, 0.13 mmol) and Et_3N (119 mg, 1.85 mmol) in CH_2Cl_2 (160 mL) at -30 °C. The solution was stirred under N_2 for 12 h. The organic layer was washed with water (3 × 50 mL) and dried over anhydrous Na_2SO_4. The crude material was purified using column chromatography (eluent: CH_2Cl_2/MeOH = 30/1, v/v) to afford compound **2a** as a yellow solid (183 mg, 53%). Its NMR data were identical to those of compound **2a** synthesized via a [1+1] one-pot approach.

Synthesis of macrocycle **2b**

Following a procedure similar to that described for compound **2a** using compound **13'c** and **12a** as the starting material, **2b** was obtained as an orange solid in 60% yield. Due to the extremely poor solubility of compound **2b**, NMR data could not be obtained. ESI-HRMS m/z: $[C_{76}H_{101}N_8O_{12}]^+$ 1317.7530; 1317.7534 was found.

Synthesis of macrocycle **2c**

Following a procedure similar to that described for compound **2a** using compound **13'b** and **12b** as the starting material, **2c** was obtained as an orange solid in 36% yield. ^{1}H NMR (400 MHz, $CDCl_3$): δ 9.67 (s, 4H), 9.50 (s, 2H), 9.32 (d, *J* = 2.3 Hz, 4H), 7.64 (dd, *J* = 8.7, 2.3 Hz, 4H), 6.98 (d, *J* = 8.7 Hz, 4H), 6.55 (s, 2H), 4.25-4.12 (m, 15H), 2.11-2.07 (m, 4H), 1.86-1.77 (m, 12H), 1.52-1.13 (m, 96H), 1.01 (d, *J* = 6.2 Hz, 24H), 0.86-0.81 (m, 24H). ^{13}C NMR (100 MHz, $CDCl_3$/CD_3OD, 9:1, v/v) δ: 162.9, 160.3, 150.4, 146.7, 140.7, 128.7, 118.4, 118.0, 116.1, 111.0, 98.3, 73.5, 67.8, 48.7, 48.5, 38.1, 37.5, 32.0, 31.9, 31.4, 30.2, 29.9, 29.7, 29.4, 26.9, 26.8, 25.4, 22.8, 22.7, 22.6, 14.13, 14.1. ESI-HRMS m/z: $[C_{124}H_{198}N_8O_{12}]^{2+}$ 996.2573; 996.2576 was found.

Synthesis of macrocycle **2d**

Following a procedure similar to that described for compound **2a** using compound **13'd** and **12b** as the starting material, **2d** was obtained as an orange solid in 56% yield. ^{1}H NMR (400 MHz, $CDCl_3$/CD_3OD, 9:1, v/v): δ 9.82 (s, 4H), 9.39 (s, 2H), 9.34-9.23 (m, 4H), 7.65 (dd, *J* = 8.2, 2.0 Hz, 4H), 7.01 (d, *J* = 8.2 Hz, 5H), 6.56 (s, 2H), 4.42-4.17 (m, 16H), 1.99-1.72 (m, 24H), 1.09-0.91 (m, 48H). ^{13}C NMR (100 MHz, $CDCl_3$/CD_3OD, 9:1, v/v) δ: 164.7, 161.2, 147.3, 129.1, 119.5, 117.3, 113.2, 69.4, 65.9, 47.9, 47.4, 38.0, 37.2, 27.7, 25.2, 25.1, 22.8, 19.6, 13.1. ESI-HRMS m/z: $[C_{80}H_{110}N_8O_{12}]^{2+}$ 687.4112; 687.4116 was found.

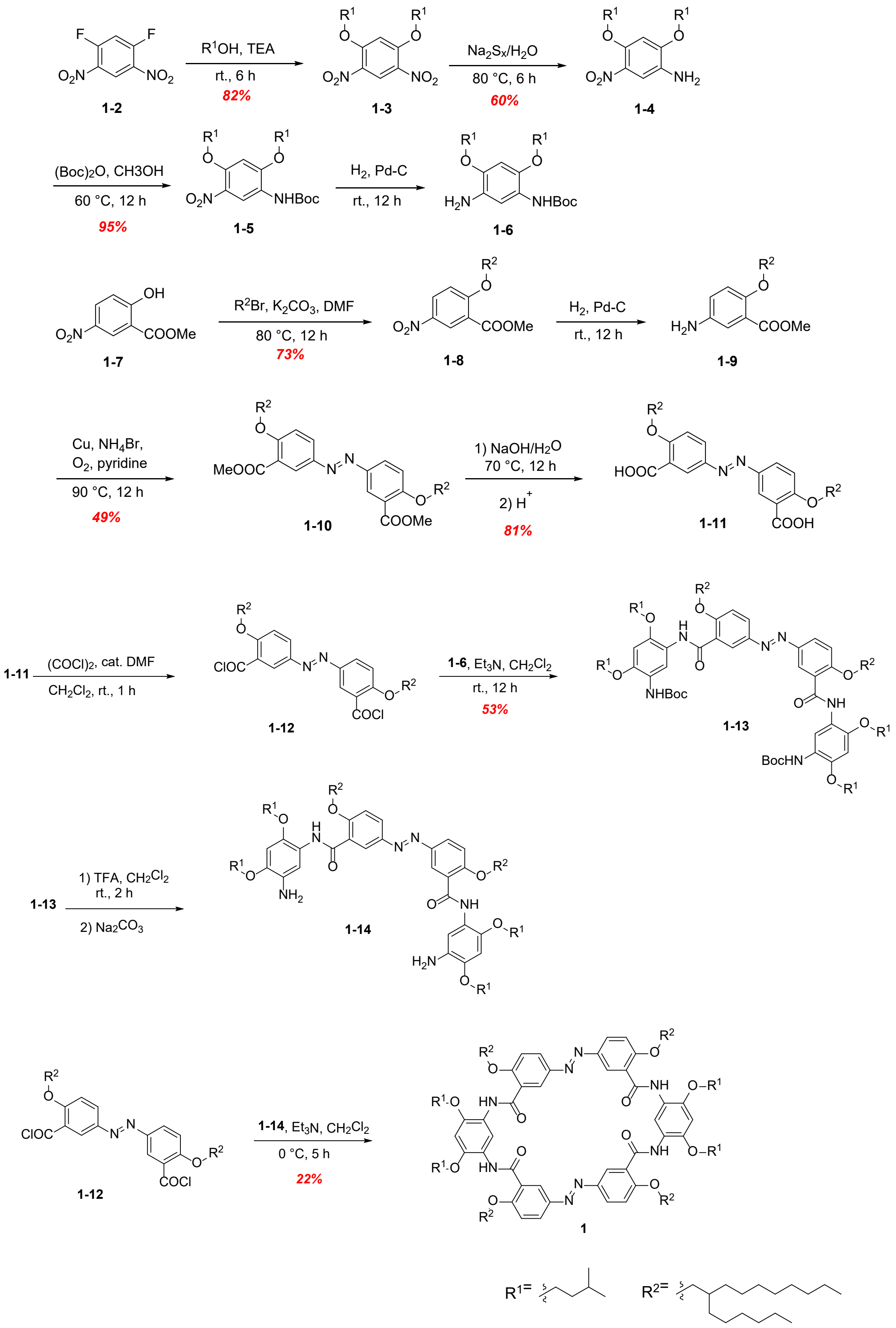

Scheme S2. Synthetic route of azo-macrocycle **1**

2.2 Synthesis of macrocycle 1

Compounds **1-2** to **1-14** were synthesized according to similar procedures in the literature [1].

Synthesis of macrocycle **1**

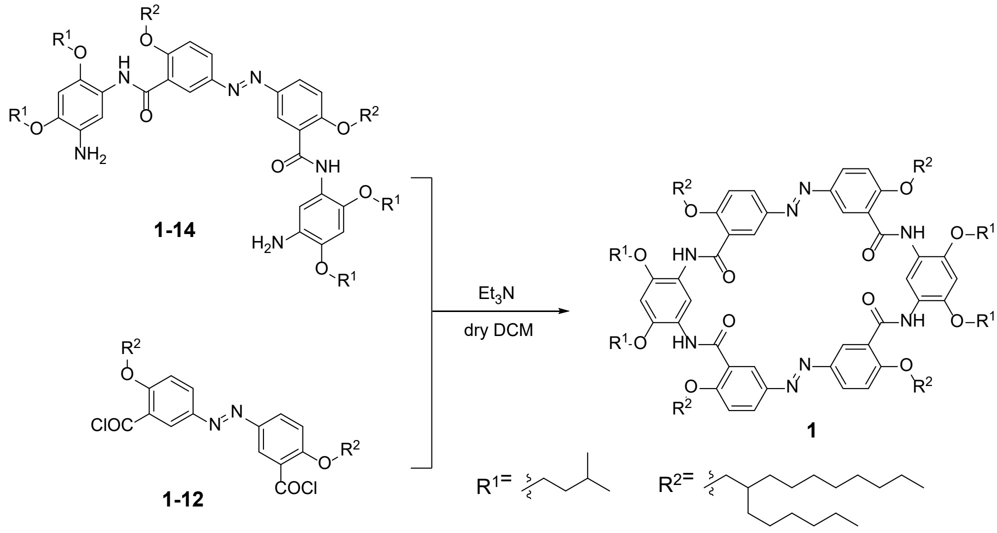

Compound **1-12** (195 mg, 0.25 mmol) in CH_2Cl_2 (30 mL) was added dropwise to the solution of **1-14** (300 mg, 0.24 mmol) and Et_3N (95.0 mg, 0.94 mmol) in CH_2Cl_2 (100 mL) at 0 °C. The solution was stirred under N_2 for 5 h. The organic layer was washed with water (3 × 50 mL) and dried over anhydrous Na_2SO_4. The crude material was purified using column chromatography (eluent: CH_2Cl_2/MeOH = 100/1, v/v) to produce an orange residue, which was dissolved in a small amount of CH_2Cl_2, adding a large amount of acetonitrile to yield the macrocycle **1** as an orange solid (100 mg, 22%). ^{1}H NMR (400 MHz, $CDCl_3$): δ 9.90 (s, 2H), 9.71 (s, 4H), 9.22 (d, *J* = 2.7 Hz, 4H), 7.99 (dd, *J* = 8.8, 2.7 Hz, 4H), 7.09 (d, *J* = 8.8 Hz, 4H), 6.54 (s, 2H), 4.19 (d, *J* = 6.8 Hz, 8H), 4.10 (t, *J* = 6.8 Hz, 8H), 2.10 (p, *J* = 6.3 Hz, 4H), 1.85-1.72 (m, 12H), 1.46-1.22 (m, 96H), 0.98 (d, *J* = 6.3 Hz, 24H), 0.87-0.82 (m, 24H).

2.3 Characterizations

2.3.1 NMR spectra

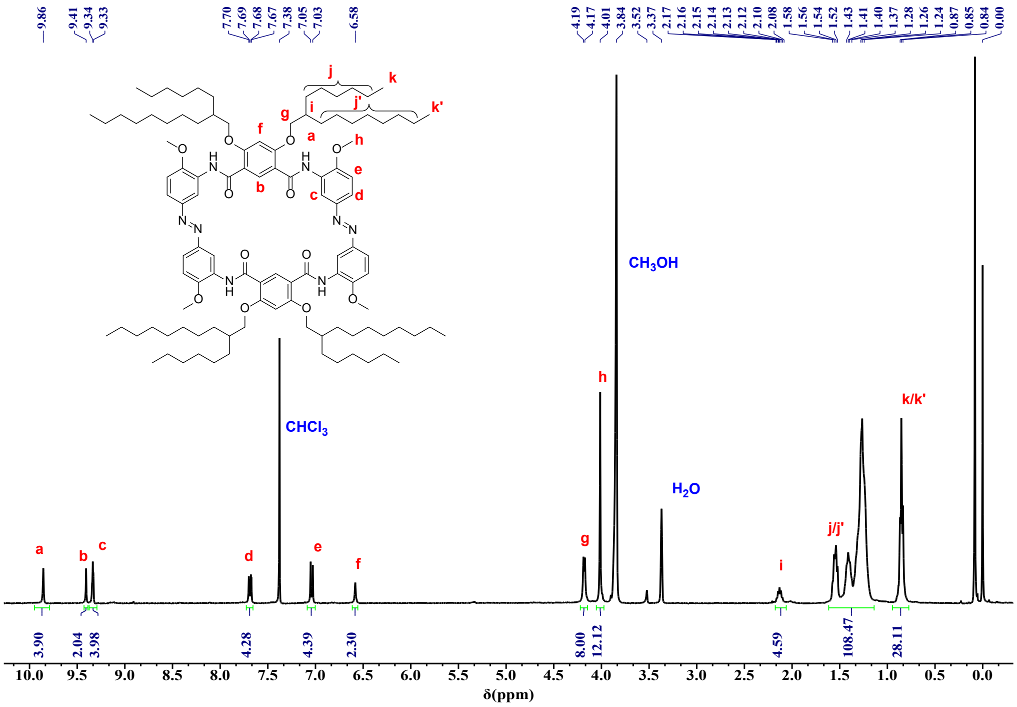

Figure S1. ^{1}H NMR spectrum (400 MHz, 298 K, $CDCl_3/CD_3OD$, 9:1, v/v) of **2a**.

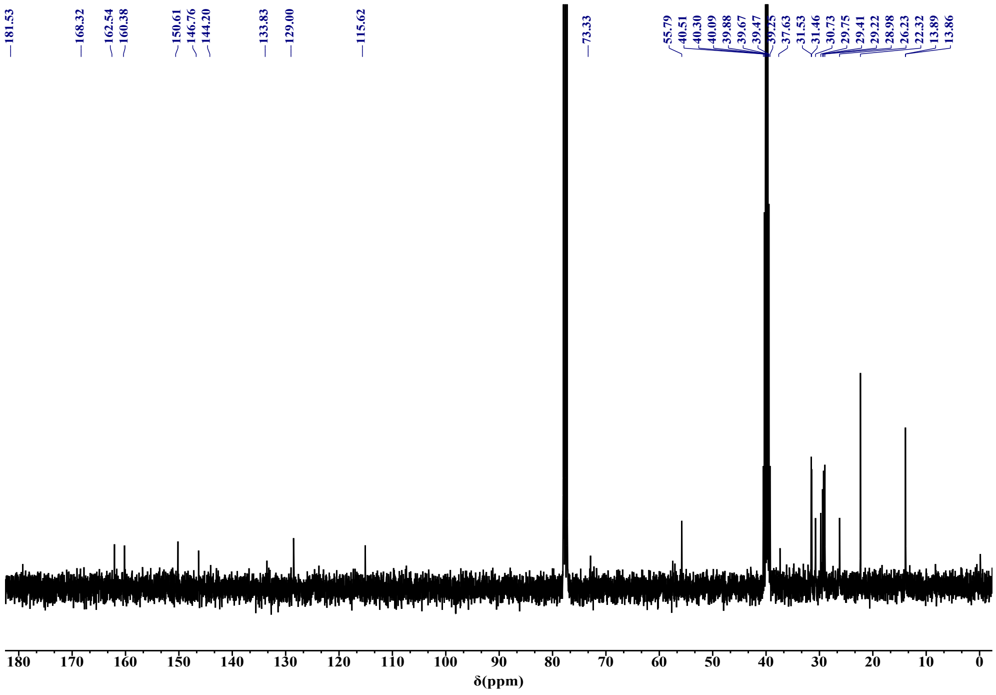

Figure S2. ^{13}C NMR spectrum (100 MHz, 298 K, $CDCl_3$/DMSO-d_6, 5:1, v/v) of **2a**.

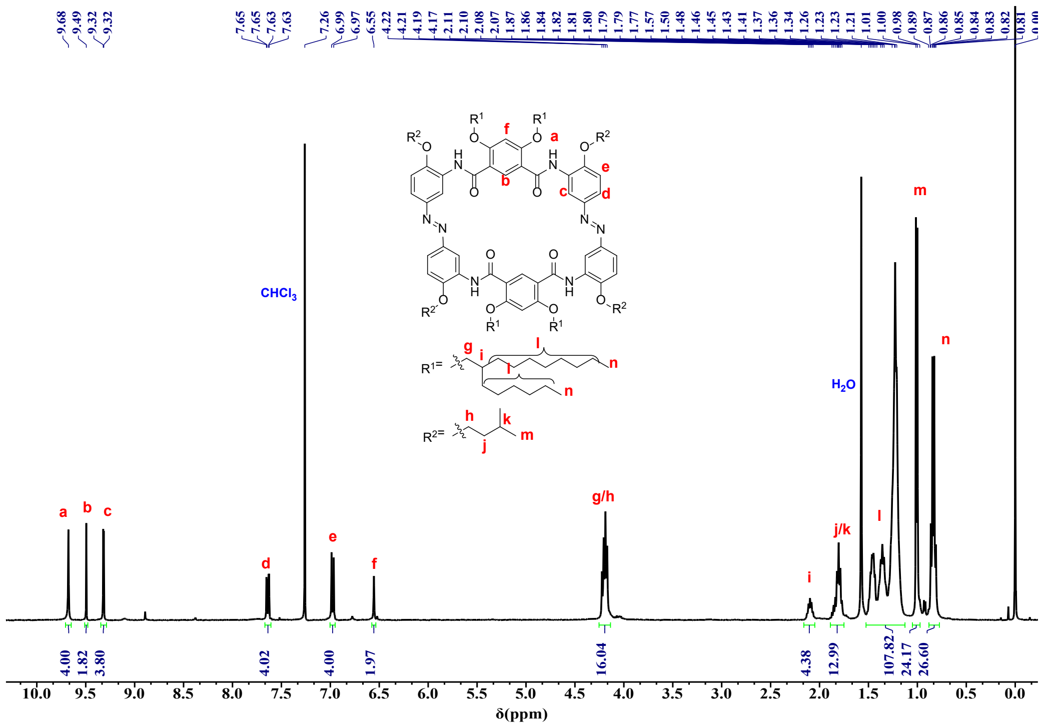

Figure S3. ^{1}H NMR spectrum (400 MHz, 298 K, $CDCl_3$) of **2c**.

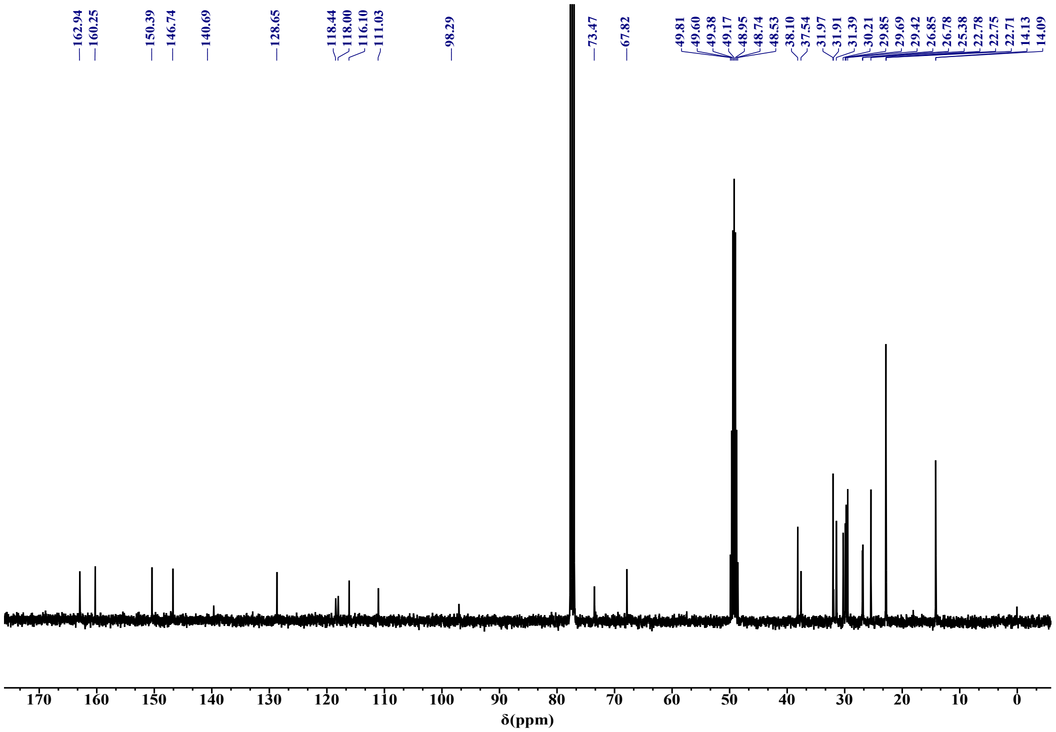

Figure S4. ^{13}C NMR spectrum (100 MHz, 298 K, $CDCl_3/CD_3OD$, 9:1, v/v) of **2c**.

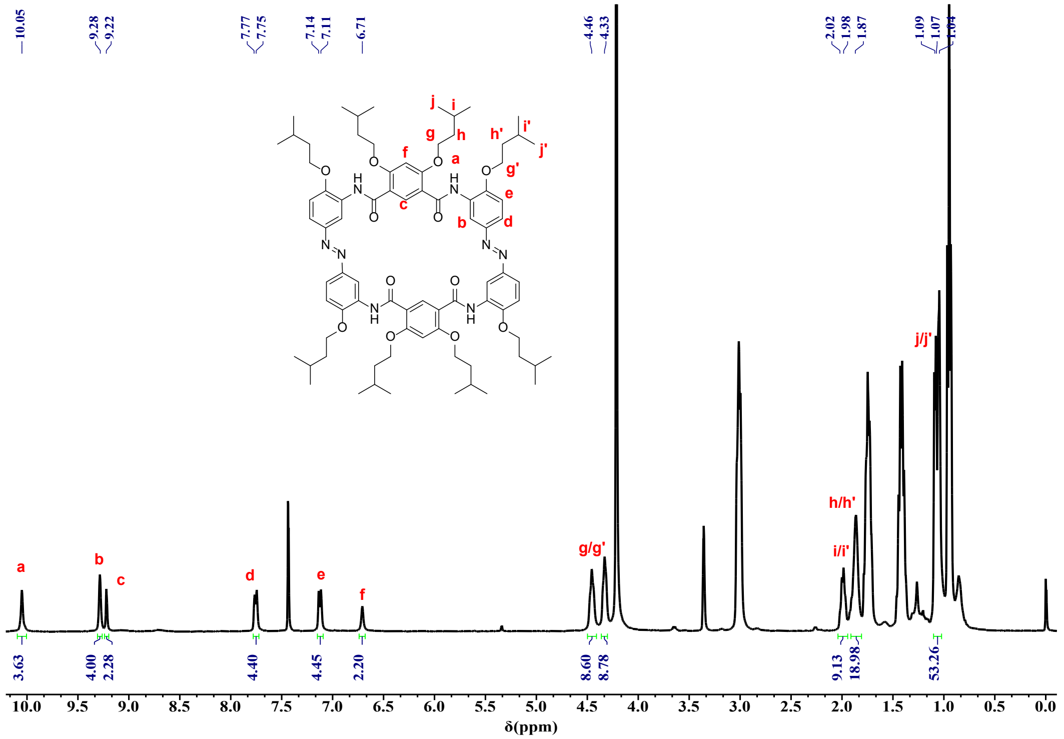

Figure S5. ^{1}H NMR spectrum (400 MHz, 298 K, $CDCl_3$/CD_3OD, 9:1, v/v) of **2d** + excess dibutylamine hydrochloride.

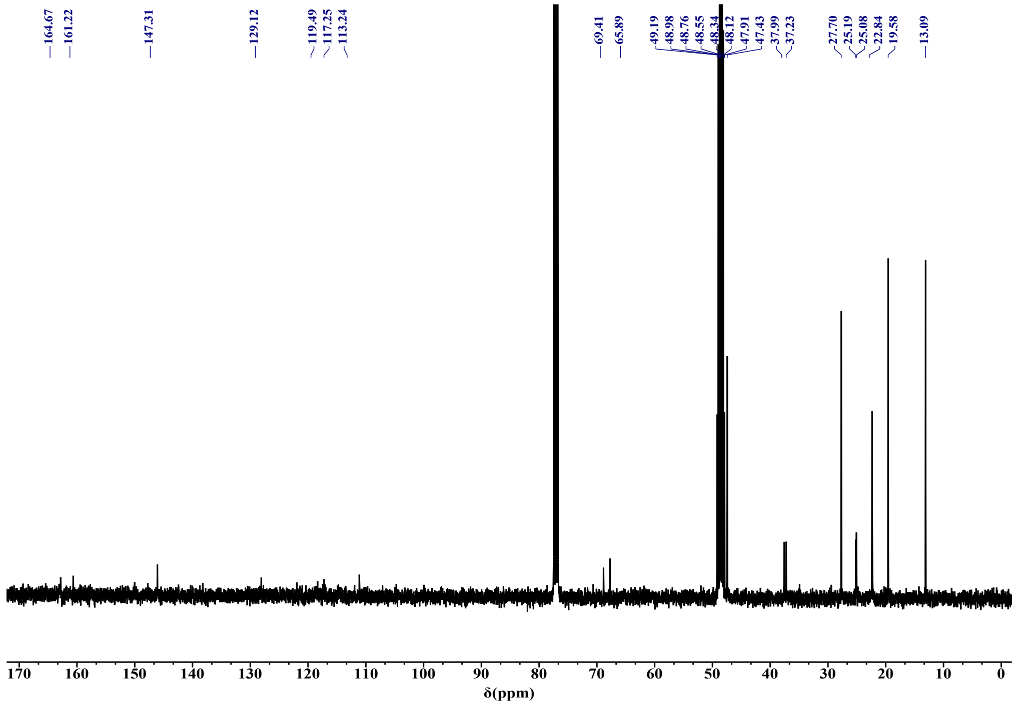

Figure S6. ^{13}C NMR spectrum (100 MHz, 298 K, $CDCl_3$/CD_3OD, 9:1, v/v) of **2d** + excess dibutylamine hydrochloride.

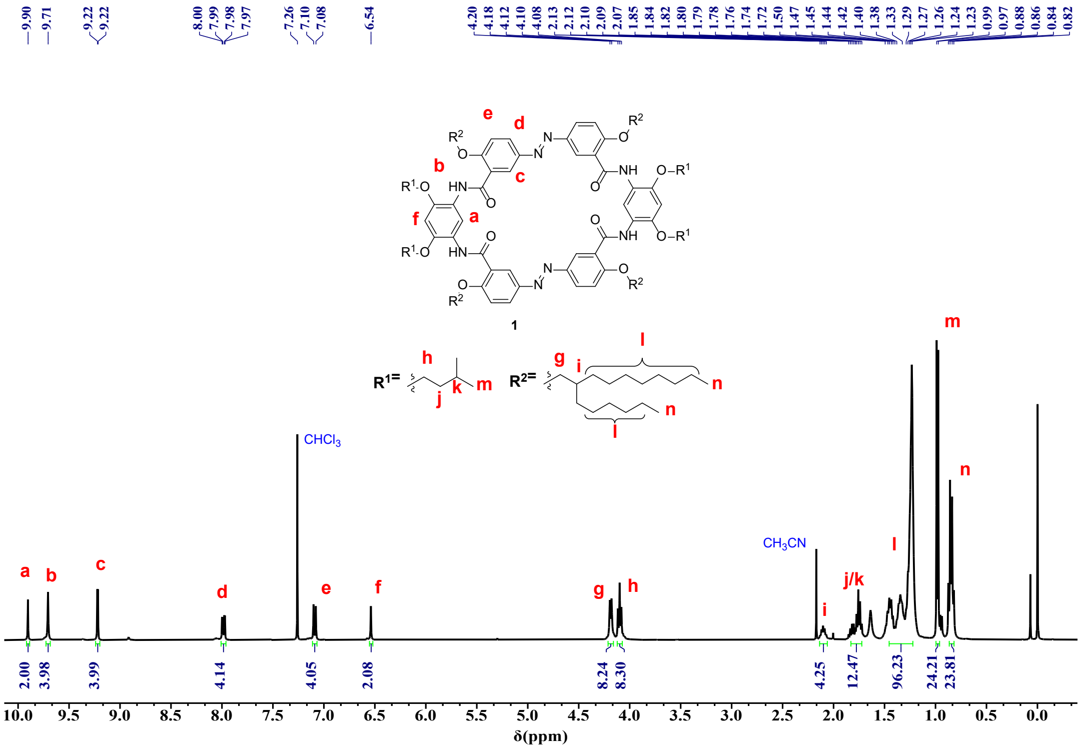

Figure S7. ^{1}H NMR spectrum (400 MHz, 298 K, $CDCl_3$) of **1**.

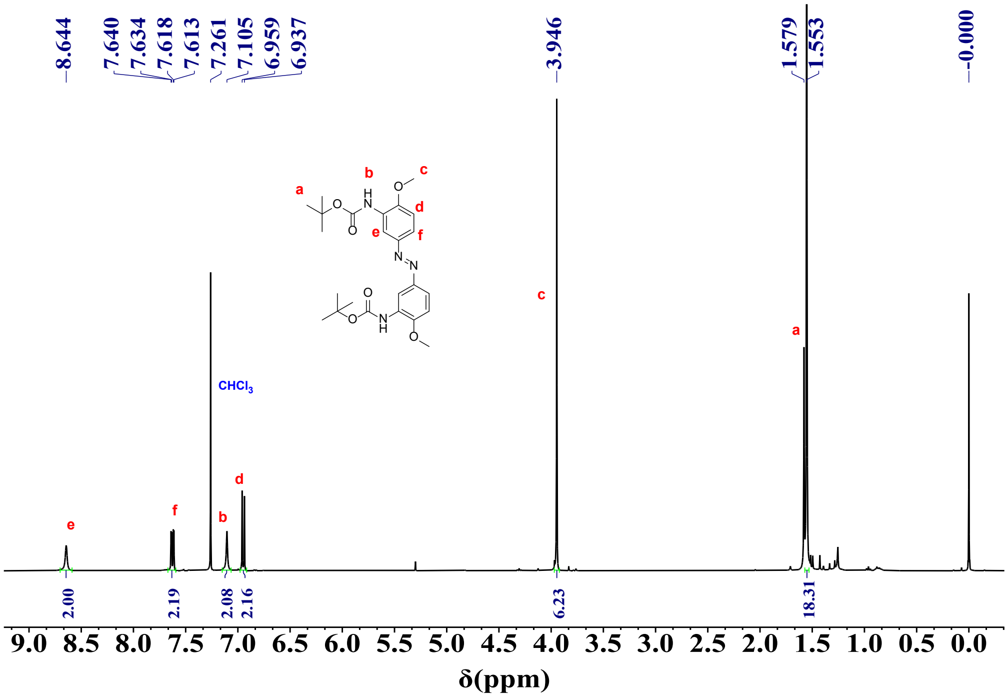

Figure S8. ^{1}H NMR spectrum (400 MHz, 298 K, $CDCl_3$) of **11a**.

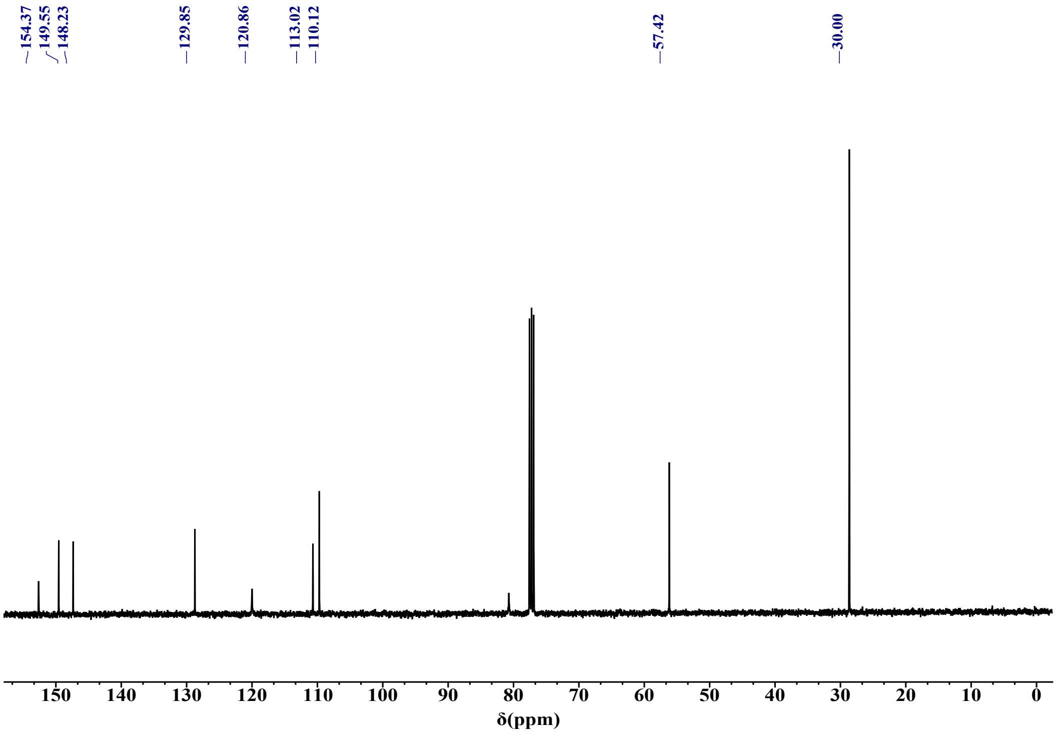

Figure S9. ^{13}C NMR spectrum (100 MHz, 298 K, $CDCl_3$) of **11a**.

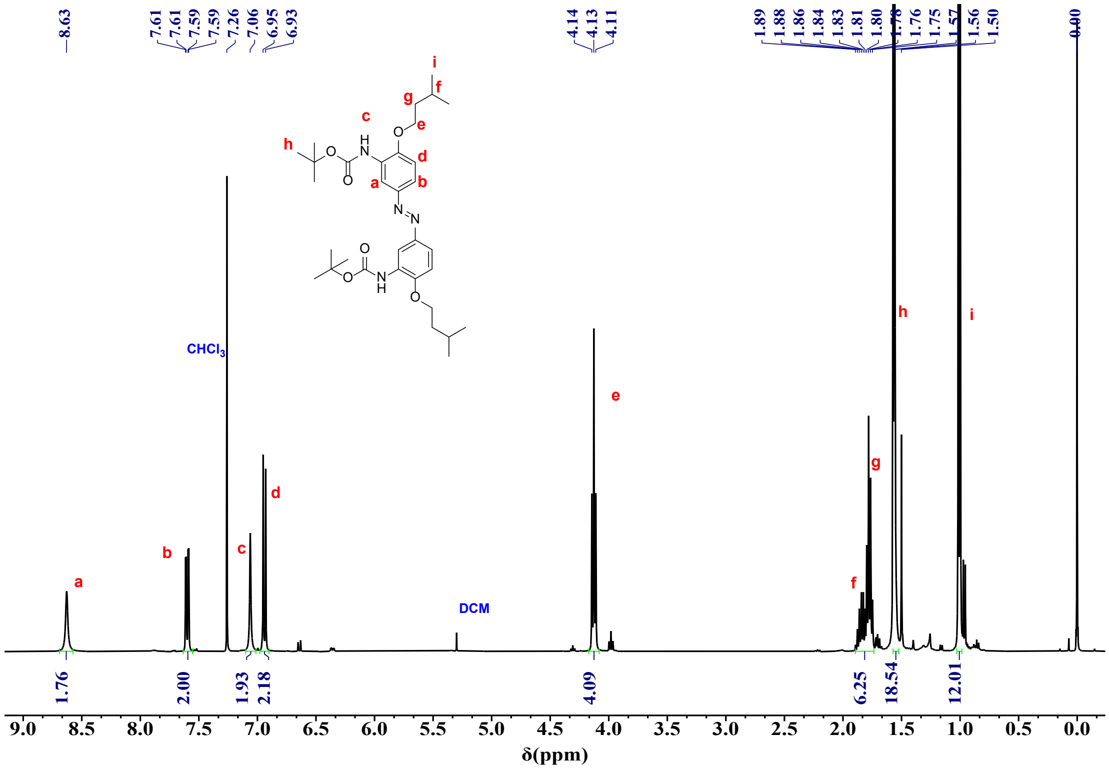

Figure S10. ^{1}H NMR spectrum (400 MHz, 298 K, $CDCl_3$) of **11b**.

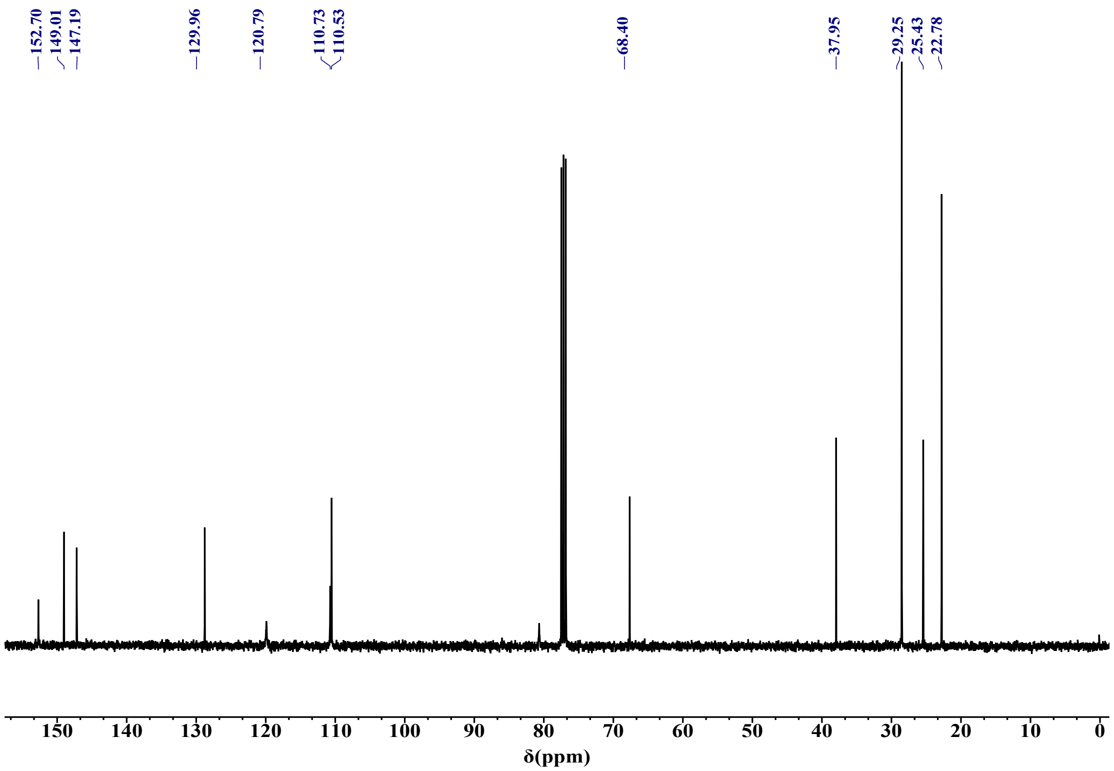

Figure S11. ^{13}C NMR spectrum (100 MHz, 298 K, $CDCl_3$) of **11b**.

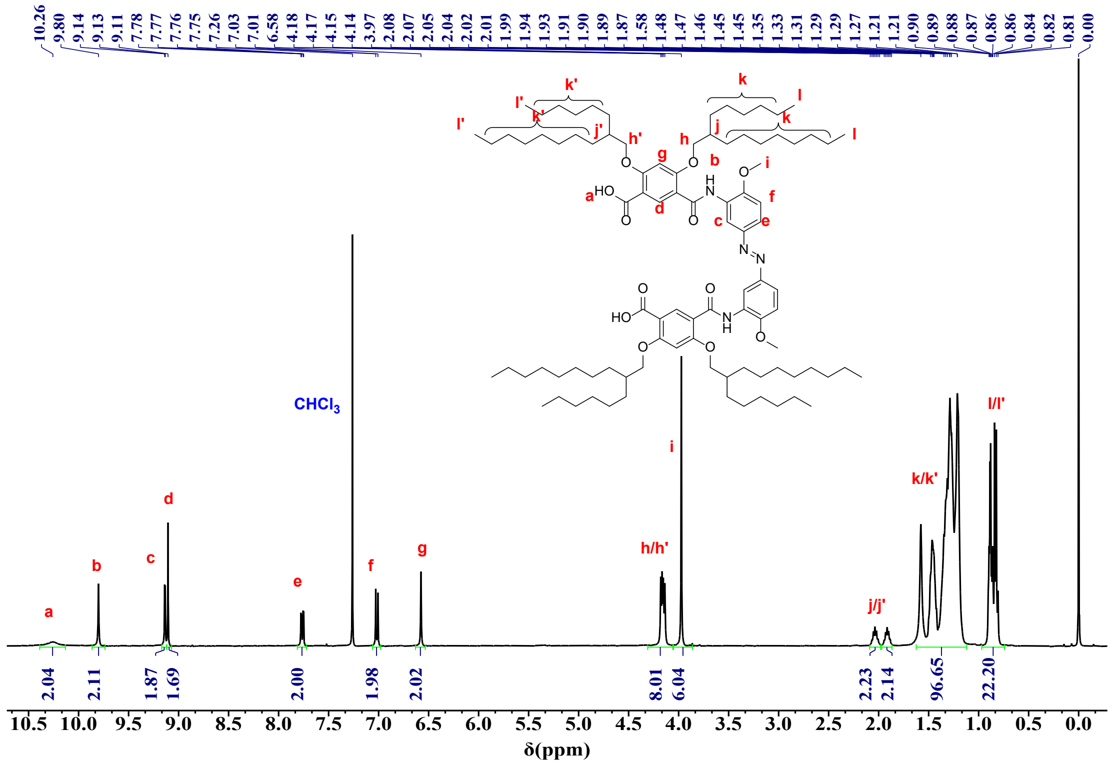

Figure S12. ^{1}H NMR spectrum (400 MHz, 298 K, $CDCl_3$) of **13a**.

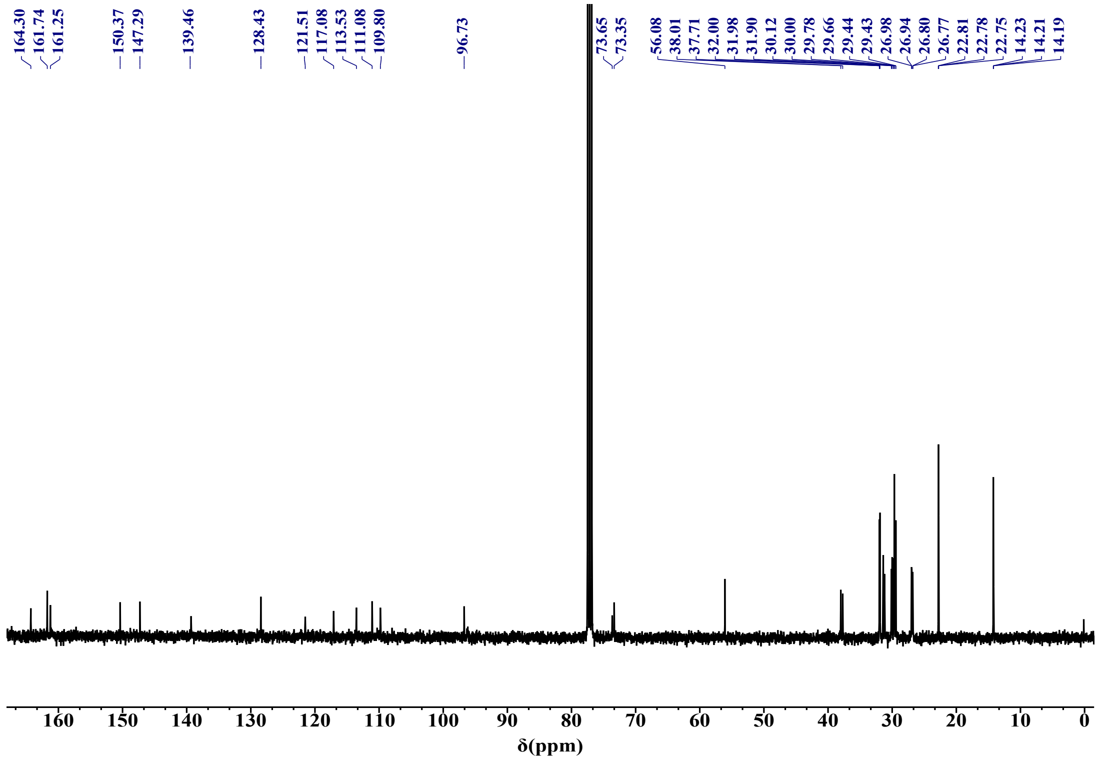

Figure S13. ^{13}C NMR spectrum (100 MHz, 298 K, $CDCl_3$) of **13a**.

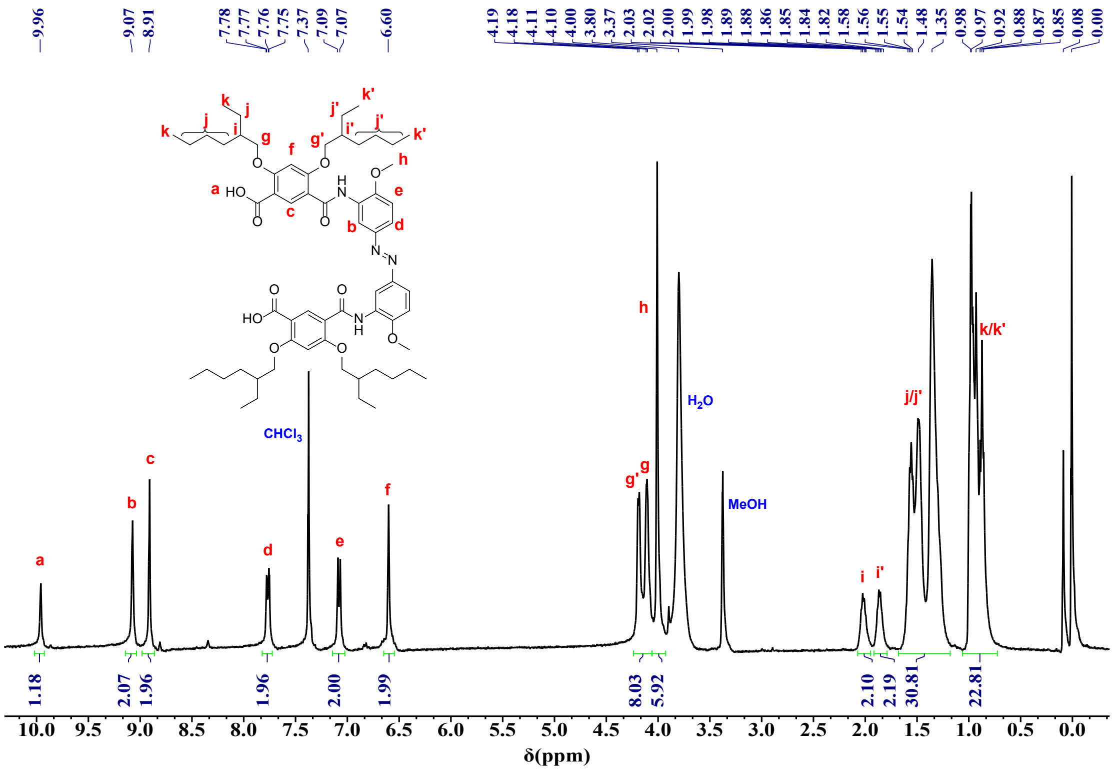

Figure S14. ^{1}H NMR spectrum (400 MHz, 298 K, $CDCl_3/CD_3OD$, 9:1, v/v) of **13b**.

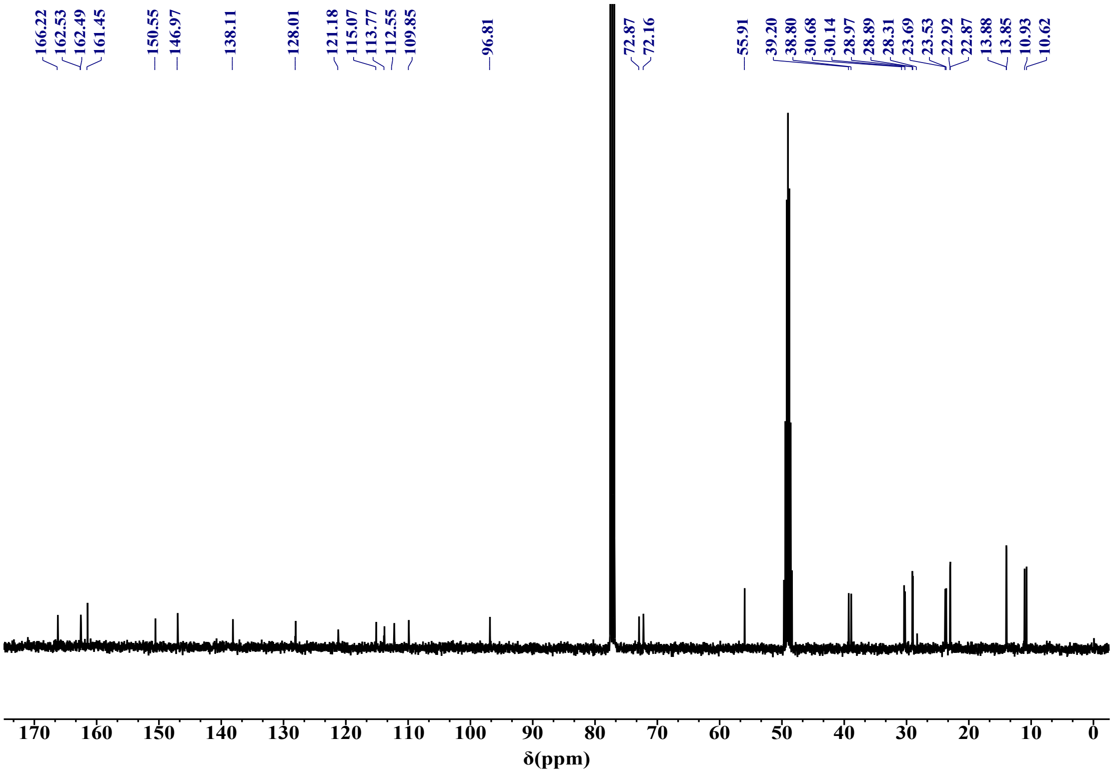

Figure S15. ^{13}C NMR spectrum (100 MHz, 298 K, $CDCl_3/CD_3OD$, 9:1, v/v) of **13b**.

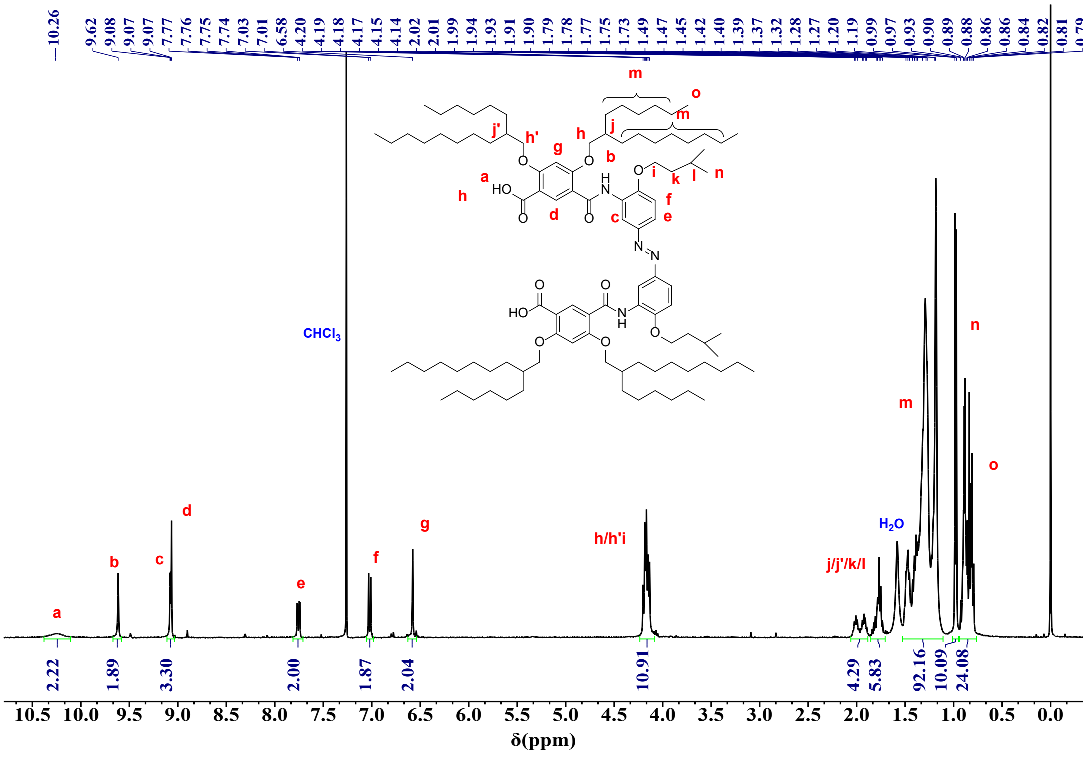

Figure S16. ^{1}H NMR spectrum (400 MHz, 298 K, $CDCl_3$) of **13c**.

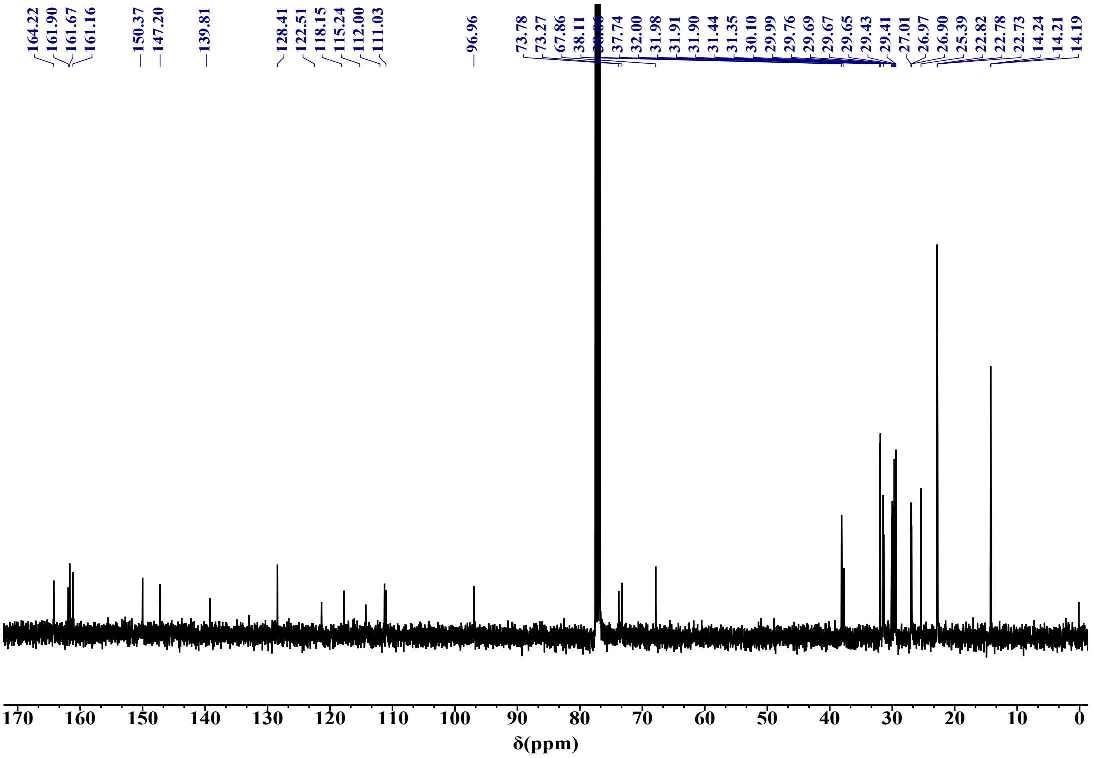

Figure S17. ^{13}C NMR spectrum (100 MHz, 298 K, $CDCl_3$) of **13c**.

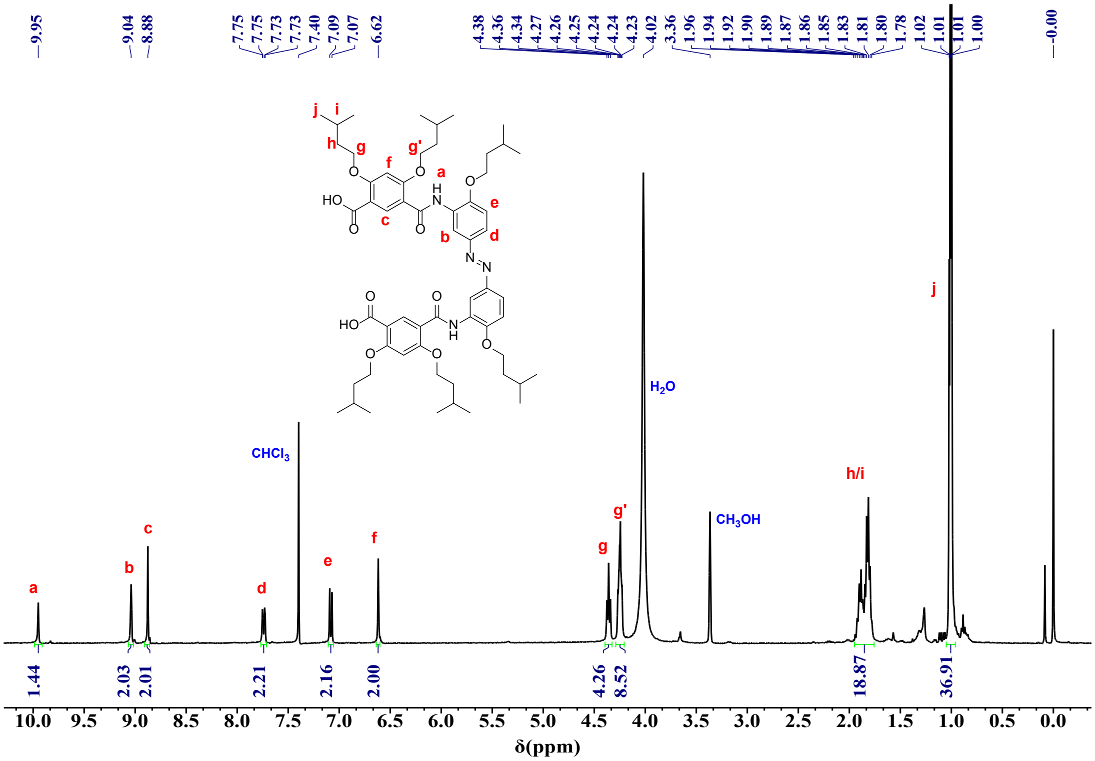

Figure S18. ^{1}H NMR spectrum (400 MHz, 298 K, $CDCl_3/CD_3OD$, 9:1, v/v) of **13d**.

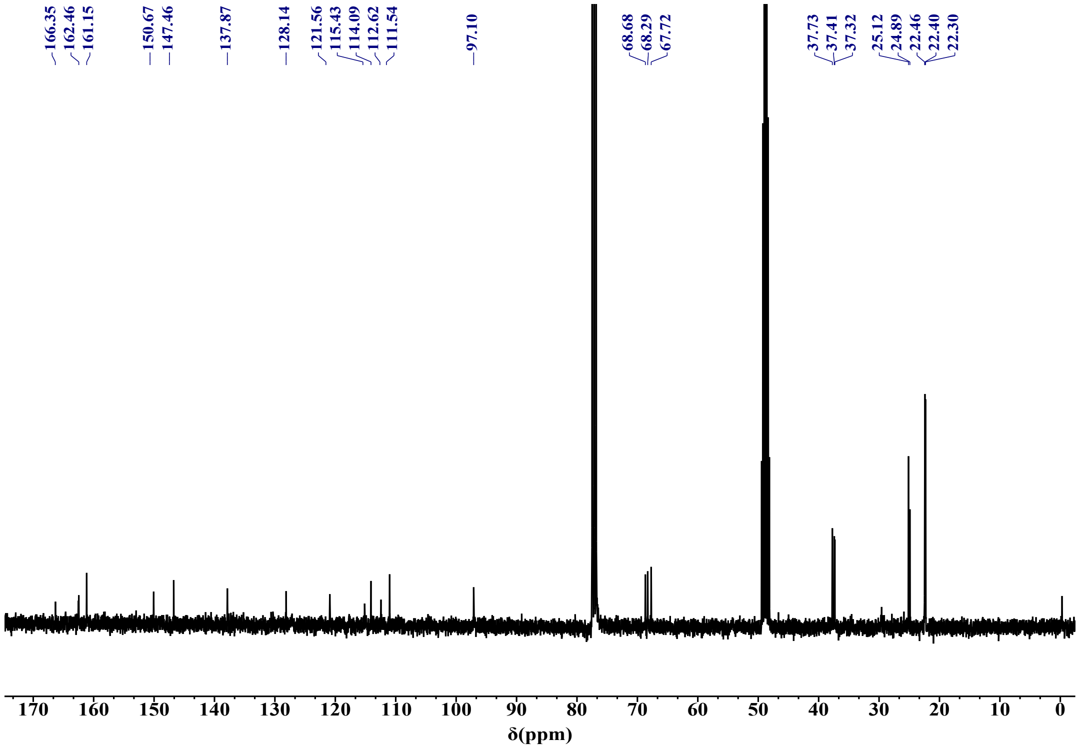

Figure S19. ^{13}C NMR spectrum (100 MHz, 298 K, $CDCl_3/CD_3OD$, 9:1, v/v) of **13d**.

p.s. It is unable to obtain satisfactory NMR spectra for **2b** due to its extremely poor solubility in all common deuterated solvents ($CDCl_3$, CD_3CN, DMSO-d_6, mixed solvent, etc.).

2.3.2 Mass spectra

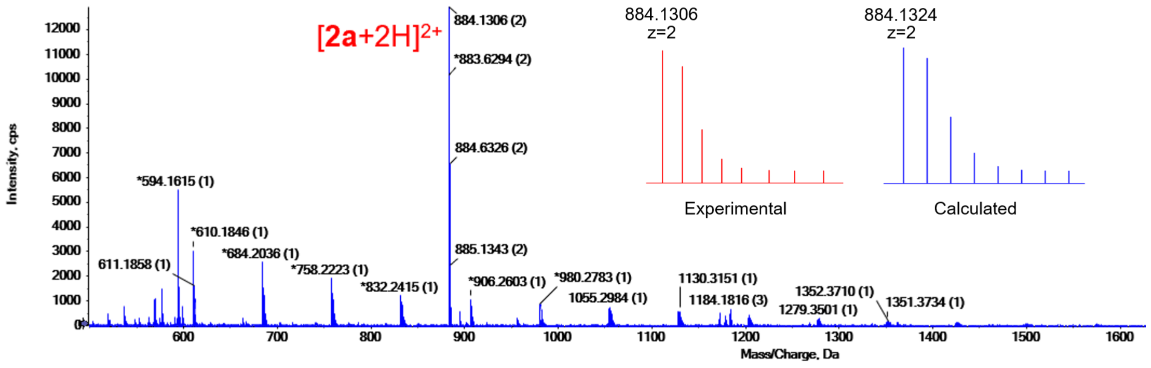

Figure S20. ESI-HRMS spectrum of **2a**. m/z: $[C_{108}H_{166}N_8O_{12}]^{2+}$ [**2a**+2H]$^{2+}$ Cal. 884.1324; 884.1306 was found

(insert: experimental (red) and simulated (blue) isotope distribution of the corresponding peak).

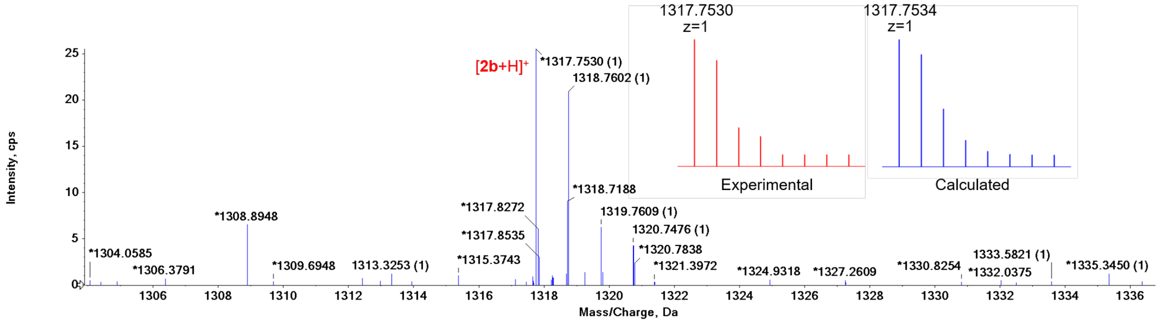

Figure S21. ESI-HRMS spectrum of **2b**. m/z: $[C_{76}H_{101}N_8O_{12}]^+$ $[\mathbf{2b}+H]^+$ Cal. 1317.7530; 1317.7534 was found (insert: experimental (red) and simulated (blue) isotope distribution of the corresponding peak).

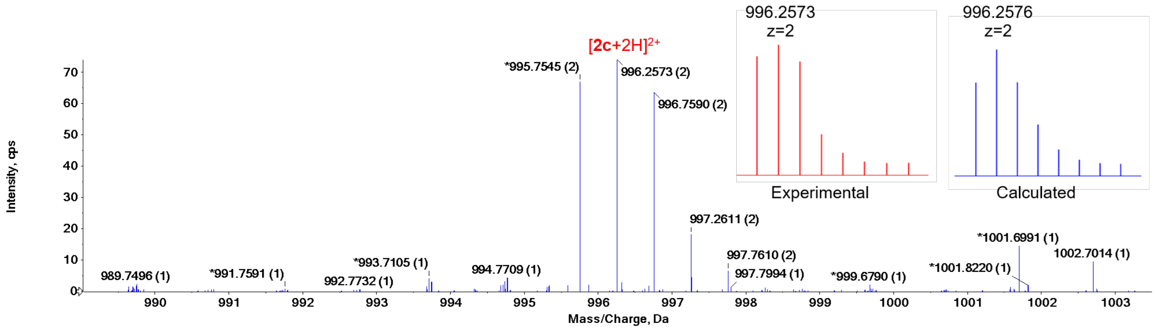

Figure S22. ESI-HRMS spectrum of **2c**. m/z: $[C_{124}H_{198}N_8O_{12}]^{2+}$ $[\mathbf{2c}+2H]^{2+}$ Cal. 996.2573; 996.2576 was found (insert: experimental (red) and simulated (blue) isotope distribution of the corresponding peak).

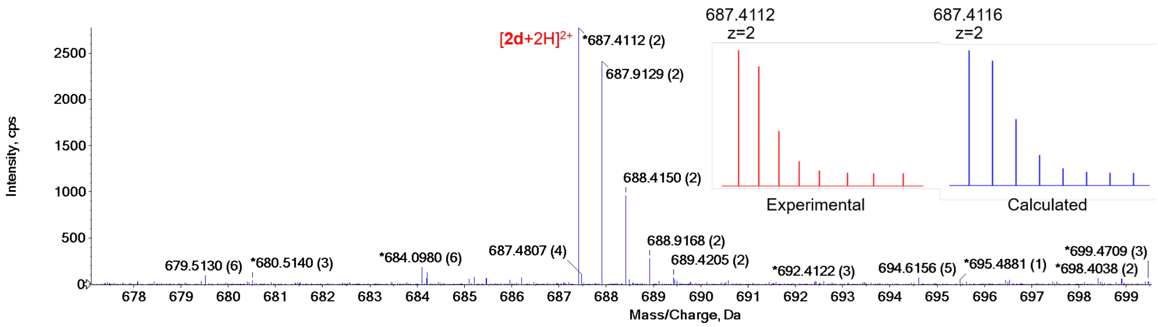

Figure S23. ESI-HRMS spectrum of **2d**. m/z: $[C_{80}H_{110}N_8O_{12}]^{2+}$ $[\mathbf{2d}+2H]^{2+}$ Cal. 687.4112; 687.4116 was found (insert: experimental (red) and simulated (blue) isotope distribution of the corresponding peak).

3. X-ray crystal structures

Crystallographic data (excluding structure factors) for macrocycle **2d** have been deposited with the Cambridge Crystallographic Data Centre (CCDC) under deposition number CCDC 2532905. Copies of the data can be obtained free of charge from the CCDC via www.ccdc.cam.ac.uk/data_request/cif. Detailed data collection and structure refinement parameters are provided in the corresponding CIF files.

Macrocycle **2d**, bearing shorter side chains, was selected for single-crystal growth. Needle-shaped yellow crystals suitable for X-ray diffraction analysis were obtained by slow evaporation of a CH_2Cl_2/CH_3OH solution of **2d** at room temperature.

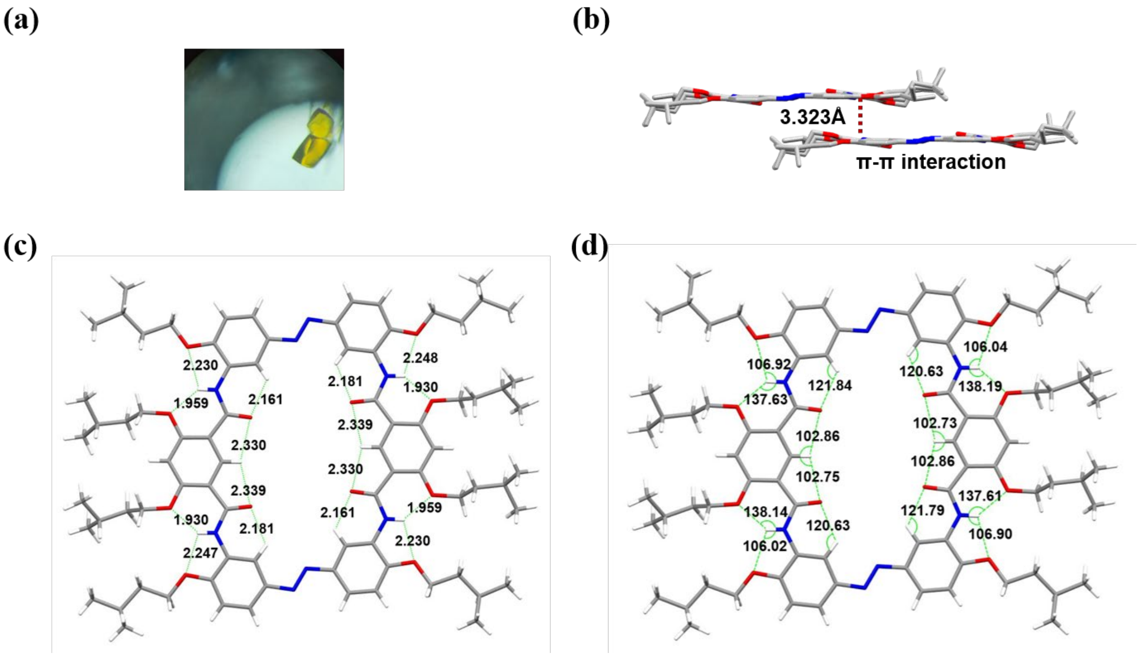

Figure S24. Crystal structure of macrocycle **2d** showing (a) the macroscopic photograph of the crystal, (b) π–π stacking interactions between two stacked macrocycles, intramolecular C-H···O and N-H···O hydrogen-bond (c) distances (Å), and (d) corresponding hydrogen-bond angles (°).

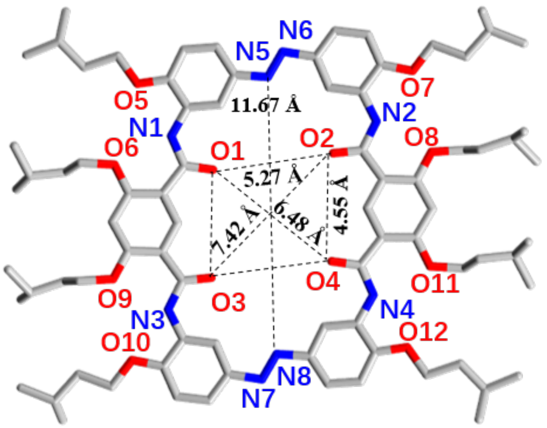

Figure S25. Single-crystal X-ray structure of macrocycle **2d** with selected interatomic distances (Å). Color code for atoms: C (gray), H (white), O (red), and N (blue).

Table S1. Crystallographic data of **2d**

Compound	**2d**
CCDC	2532905
Empirical formula	$C_{82}H_{114}Cl_6N_8O_{14}$
Formula weight	1648.51
Temperature/K	100.00(10)
Crystal system	monoclinic
Space group	$P2_1/c$
a/Å	17.6382(4)
b/Å	10.2115(2)
c/Å	23.2933(6)
α/°	90
β/°	90.975(2)
γ/°	90
Volume/Å^3	4194.81(17)
Z	2
ρ_{calc}g/cm^3	1.305
μ/mm^{-1}	0.271
F(000)	1752.0

Crystal size/mm^3	0.21 × 0.2 × 0.19
Radiation	Mo Kα (λ = 0.71073)
2θ range for data collection/°	4.224 to 58.802
Index ranges	-17≤h≤23, -12≤k≤14, -28≤l≤27
Reflections collected	32666
Independent reflections	9482 [R_{int} = 0.0275, R_{sigma} = 0.0325]
Data/restraints/parameters	9482/410/621
Goodness-of-fit on F^2	1.052
Final R indexes [I>=2σ (I)]	R_1 = 0.0629, wR_2 = 0.1778
Final R indexes [all data]	R_1 = 0.0809, wR_2 = 0.1910
Largest diff. peak/hole/e Å^{-3}	1.22/-0.91

Table S2. Intramolecular hydrogen bond parameters of **2d**.

**D—H⋯A**	**H⋯A/Å**	**D⋯A/Å**	**D—H⋯A/°**
N1-H1⋯O5	2.230	2.624	106.92
N1-H1⋯O6	1.959	2.676	137.63
N2-H2⋯O7	2.248	2.630	106.04
N2-H2⋯O8	1.930	2.651	138.19
N3-H3⋯O9	1.930	2.652	138.14
N3-H3⋯O10	2.247	2.630	106.02
N4-H4⋯O11	1.959	2.675	137.61
N4-H4⋯O12	2.230	2.624	106.90

Table S3. Selected interatomic distances (Å) of **2d**.

atoms	interatomic distances (Å)
O1-O2	5.27
O1-O3	4.55
O1-O4	6.48
O2-O3	7.42
N5-N8	11.67

4. Photoisomerization studies

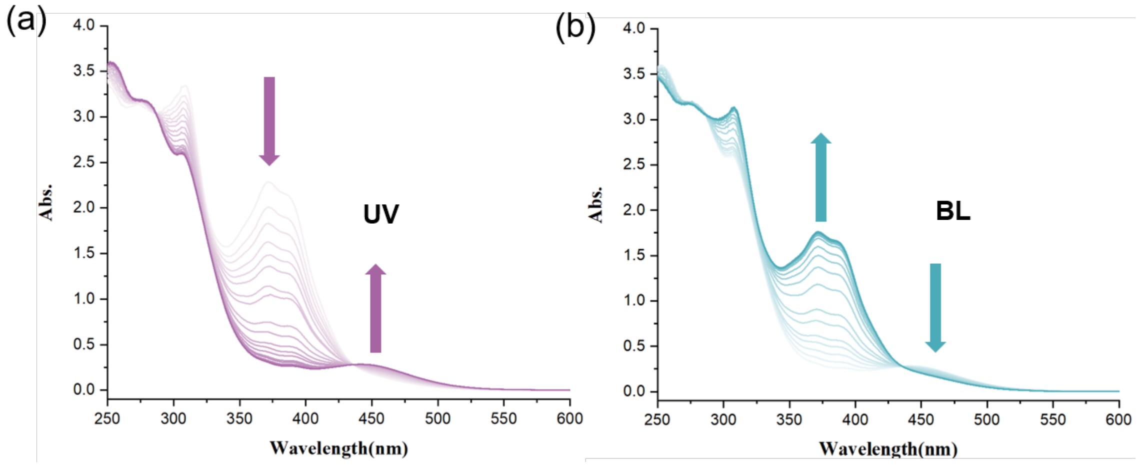

Figure S26. UV–vis absorption spectra of **2c** at 298 K (50 μM, $CHCl_3/CH_3CN$, 2:1, v/v): (a) spectral evolution upon irradiation at 365 nm (1 min) to reach the Z-rich photostationary state (PSS) and (b) spectral recovery upon irradiation at 450 nm (blue light, 3 min) to regenerate the *E*-rich photostationary state.

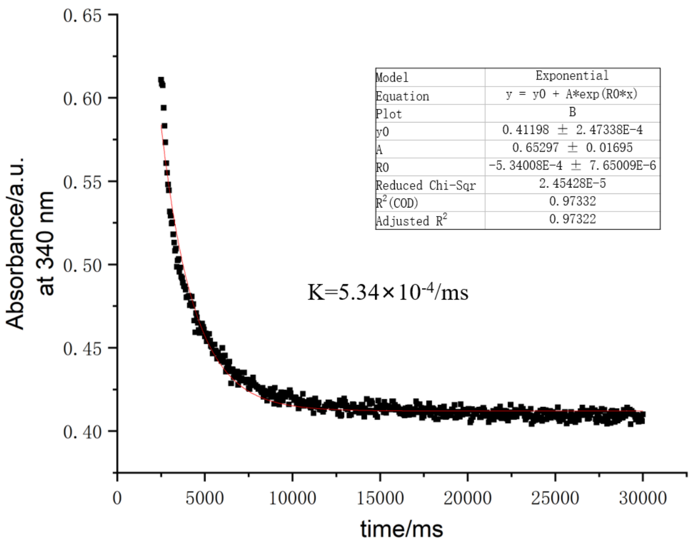

Figure S27. Photoisomerization rate of **2c** (50 μM in $CHCl_3/CH_3CN$, 2:1, v/v, 298 K) at UV 365 nm irradiation.

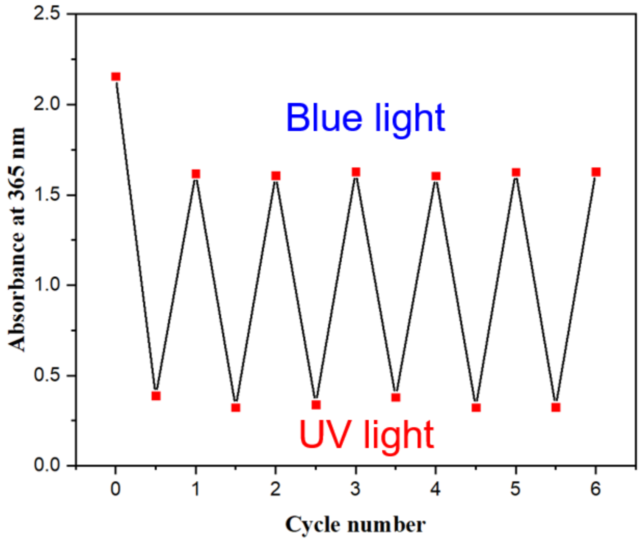

Figure S28. Absorbance intensity changes of **2c** at 298 K (50 μM, $CHCl_3/CH_3CN$, 2:1, v/v) at 365 nm upon alternating UV light and blue light (BL) irradiation.

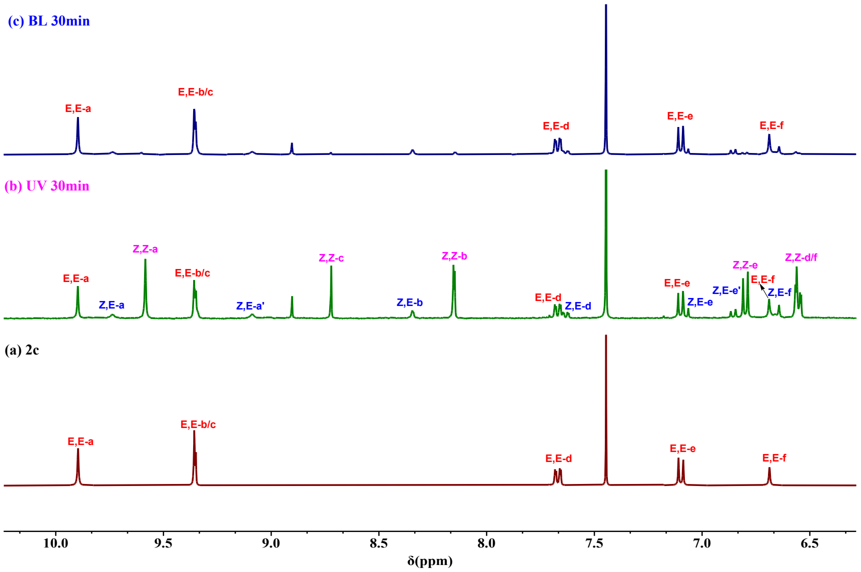

Figure S29. Stacked partial ^{1}H NMR spectra (400 MHz, 298 K, $CDCl_3/CD_3CN$, v/v, 2:1, [**2c**] = 2mM) of macrocycle **2c** before (a) and after UV irradiation for (b) 30 min, then after (c) blue light irradiation for 30 min.

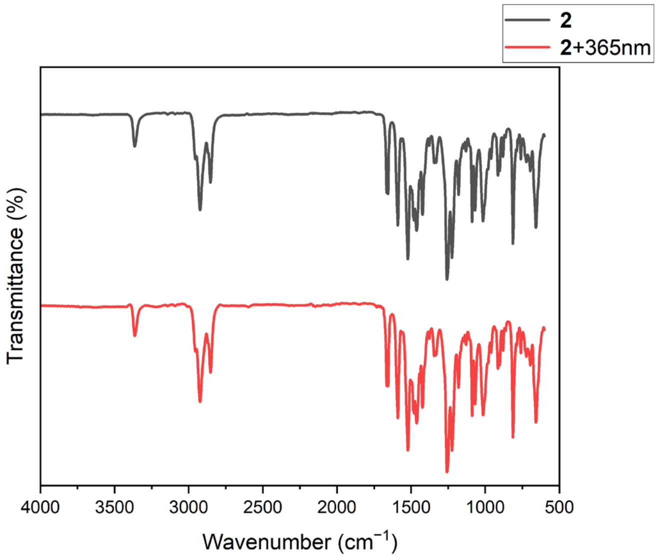

Figure S30. IR spectra of macrocycle **2** in the solid state before (black) and after (red) irradiation with 365 nm light for 30 min at 298 K. The complete overlap of the two spectra indicates that no photoisomerization occurs in the solid state under these conditions.

5. Recognition of alkali metal salts

To determine the association constants (K_a) corresponding to the reactions between macrocycle **2c** (**1**) and $LiClO_4$ ($NaClO_4$), ^1H NMR titration experiments were performed in $CDCl_3$ (298 K) solutions at a constant concentration of **2c** (**1**) (1.0 mM) and varying concentrations of $LiClO_4$ ($NaClO_4$). Association constants were obtained by a global fitting analysis to a 1:1 binding model using the website (http://supramolecular.org/).

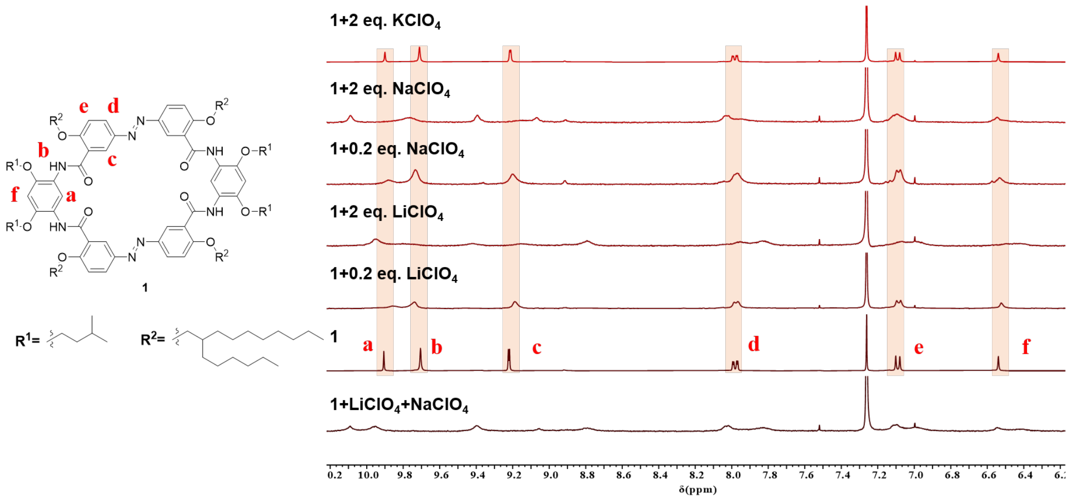

Figure S31. Partially stacked ^1H NMR spectra (400 MHz, 298 K, $CDCl_3$) of a 1.0 mM solution of **1** recorded in the absence and presence of Li^+, Na^+, and K^+ (as their ClO^{4-} salts).

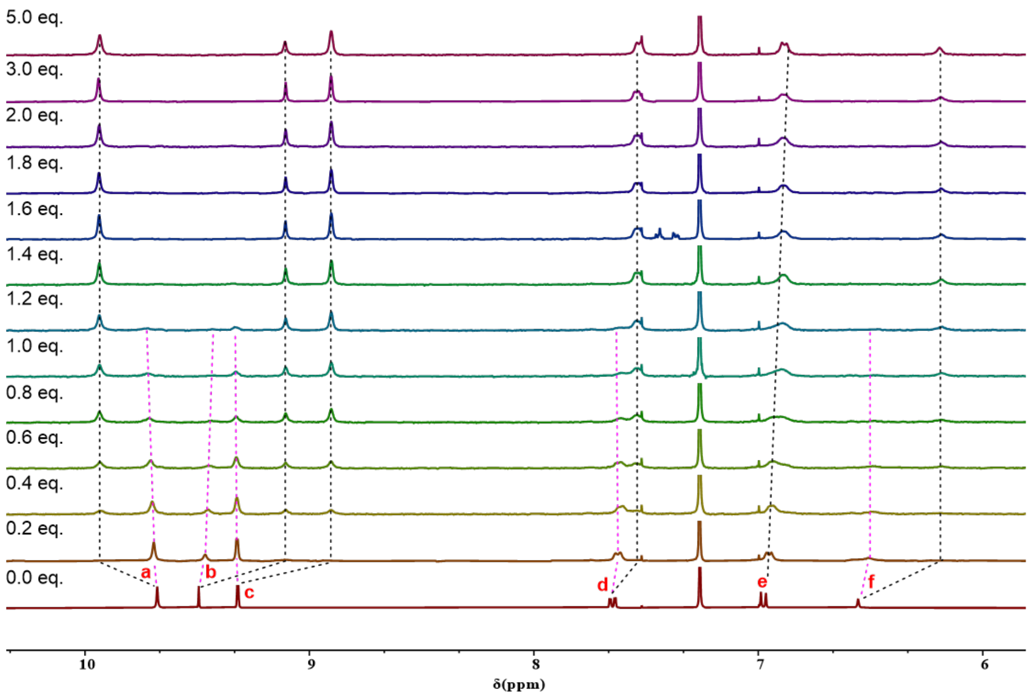

Figure S32. Partially stacked ^{1}H NMR spectra of **2c** (400 MHz, 298 K, $CDCl_3$, [**2c**] = 1.0 mM) during titration with $LiClO_4$ (0–5.0 equiv.).

Determination of association constants for host–guest association/dissociation equilibria that are slow on the NMR time scale using ^{1}H NMR spectral titrations [4]

Equilibrium: $\mathrm{A} + \mathrm{B} \underset{}{\overset{K_a}{\rightleftharpoons}} \mathrm{AB}$

Equilibrium constant: $K_a = \frac{[\mathrm{AB}]}{[\mathrm{A}][\mathrm{B}]} = \frac{[\mathrm{AB}]}{(c(\mathrm{A})-[\mathrm{AB}])(c(\mathrm{B})-[\mathrm{AB}])}$ (1)

c(A) and *c*(B) are the initial concentrations of A and B, and [A], [B], and [AB] are the equilibrium concentrations of the three species.

A and B undergo slow exchange with the complex AB on the ^{1}H NMR timescale.

Two signals for one specific proton on A can be seen in the spectrum, corresponding to complexed and uncomplexed forms of A:

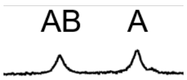

Single-point Methods

K_a is determined from the integrals of complexed and uncomplexed A. If *I*(A) denotes the integral of a signal for one specific proton of A and *I*(AB) the integral for the same proton in the complex, the concentration of AB

at equilibrium is shown by eq 2. The equilibrium expression (eq 3) is obtained after substituting it into eq (1):

$$[\mathrm{AB}] = \frac{I(\mathrm{AB})}{I(\mathrm{A})+I(\mathrm{AB})}c(\mathrm{A}) \qquad (2)$$

$$K_\mathrm{a} = \frac{I(\mathrm{AB})}{I(\mathrm{A})(c(\mathrm{B})-\frac{I(\mathrm{AB})}{I(\mathrm{A})+I(\mathrm{AB})}c(\mathrm{A}))} \qquad (3)$$

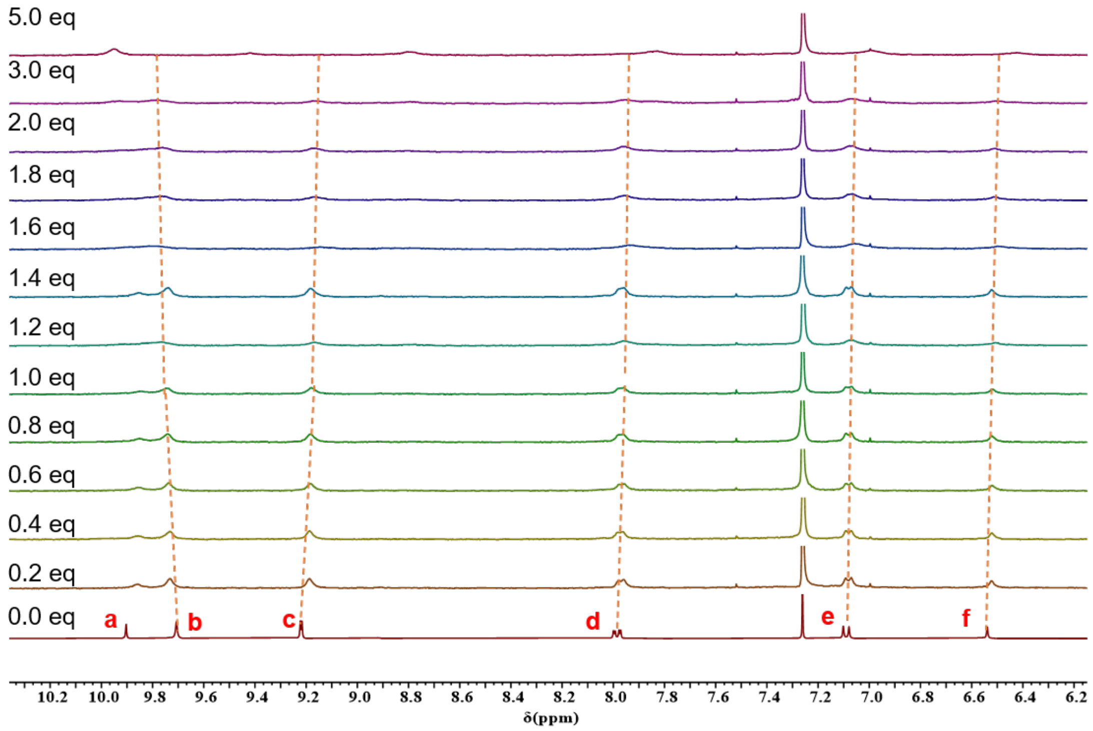

Figure S33. Partially stacked ^{1}H NMR spectra of **1** (400 MHz, 298 K, $CDCl_3$, [**1**] = 1.0 mM) during titration with $LiClO_4$ (0–5.0 equiv.).

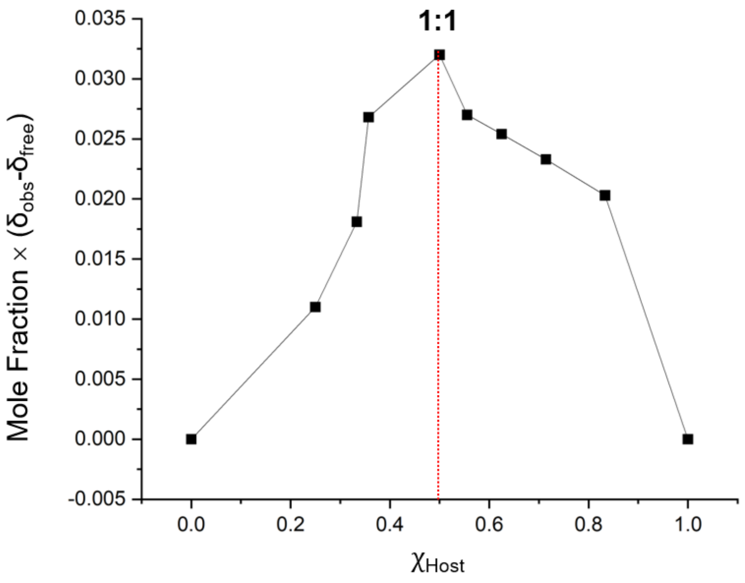

Figure S34. Job plot showing a peak maximum was reached around 0.5, corresponding to the formation of a 1:1 host–guest complex between **1** and $LiClO_4$ in $CDCl_3$ at 298 K.

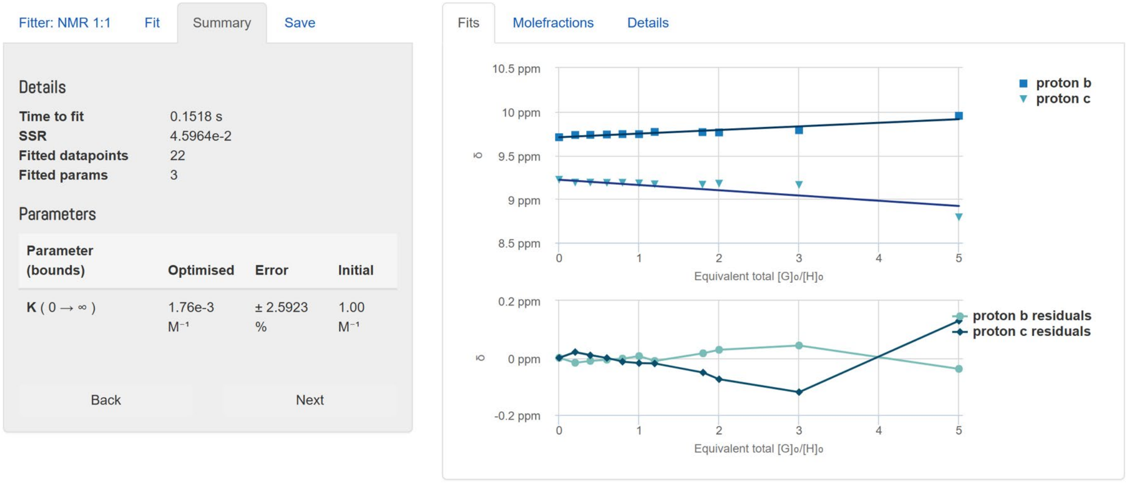

Figure S35. Nonlinear least-square analysis of the ^{1}H NMR binding data corresponding to the formation of **1** ⊃ $LiClO_4$ complex. The data extracted from Fig. S33 were fitted to a 1:1 binding model to give $K_a < 5$ M^{-1}. The residual distribution is shown below the binding isotherm. All solid lines were obtained from non-linear curve-fitting to a 1:1 binding model using the www.supramolecular.org web applet.

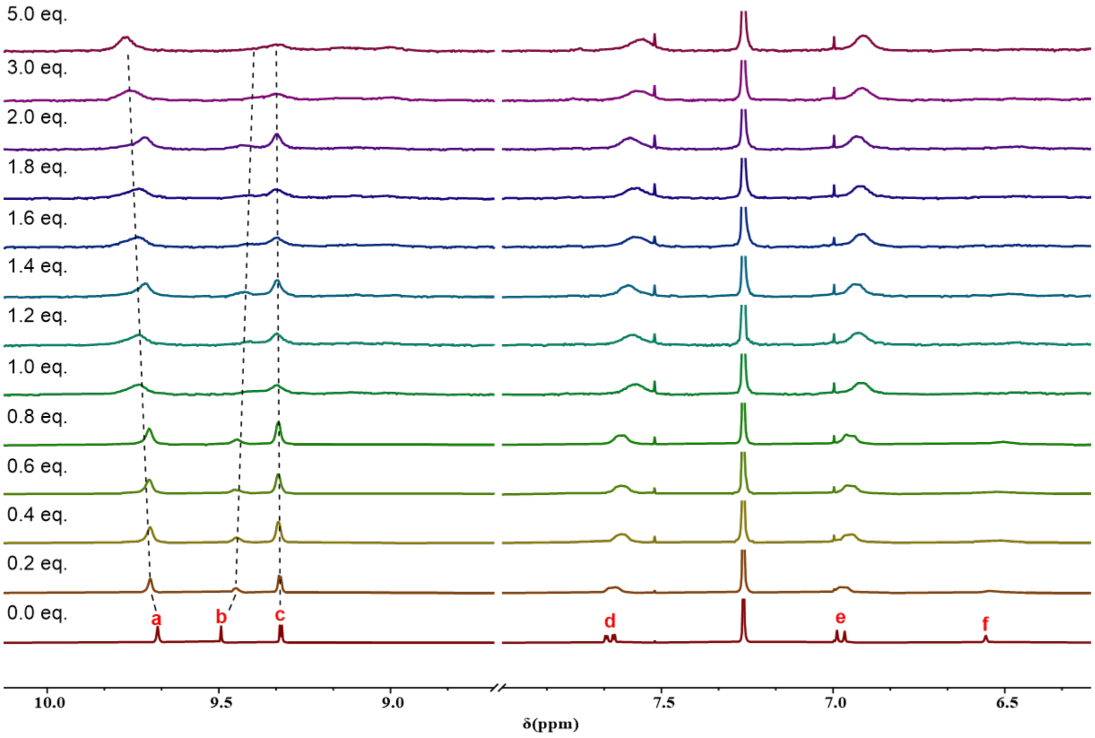

Figure S36. Partially stacked ^{1}H NMR spectra of **2c** (400 MHz, 298 K, $CDCl_3$, [**2c**] = 1.0 mM) during titration with $NaClO_4$ (0–5.0 equiv.).

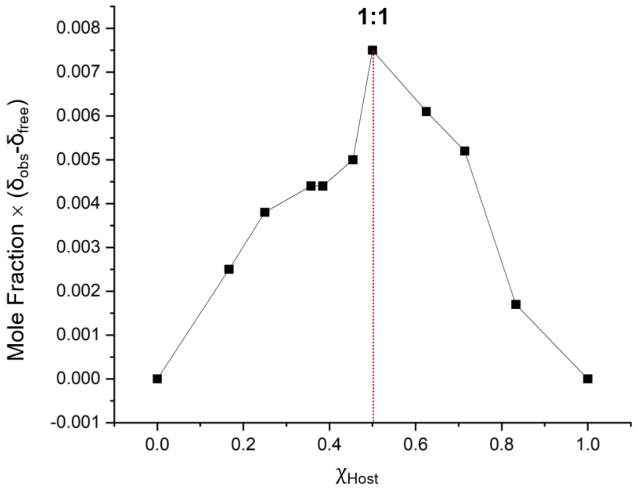

Figure S37. Job plot showing a peak maximum was reached around 0.5, corresponding to the formation of a 1:1 host–guest complex between **2c** and $NaClO_4$ in $CDCl_3$ at 298 K.

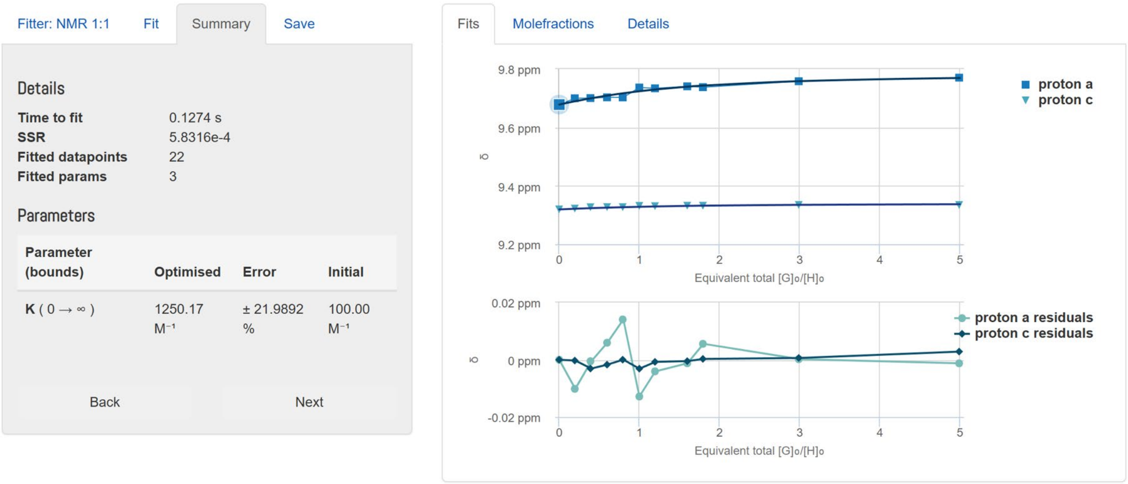

Figure S38. Nonlinear least-square analysis of the ^{1}H NMR binding data corresponding to the formation of **2c** ⊃ $NaClO_4$ complex. The data extracted from Fig. S36 were fitted to a 1:1 binding model to give K_a = (1.25 ± 0.27) × 10^3 M^{-1}. The residual distribution is shown below the binding isotherm. All solid lines were obtained from non-linear curve-fitting to a 1:1 binding model using the www.supramolecular.org web applet.

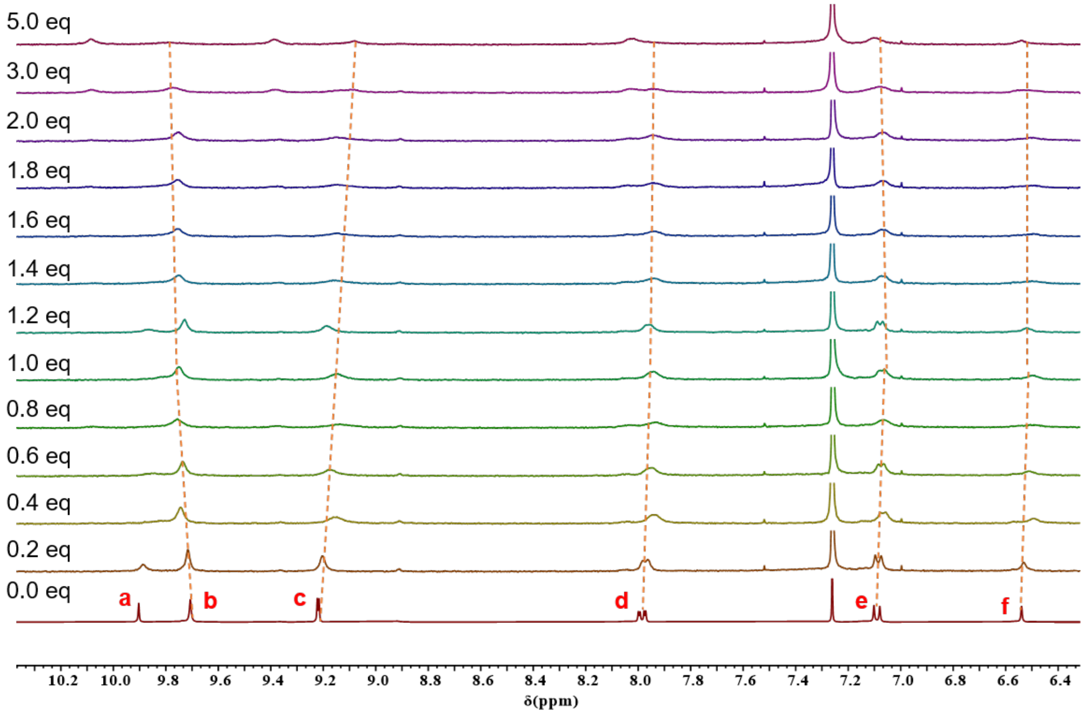

Figure S39. Partially stacked ^{1}H NMR spectra of **1** (400 MHz, 298 K, $CDCl_3$, [**1**] = 1.0 mM) during titration with $NaClO_4$ (0–5.0 equiv.).

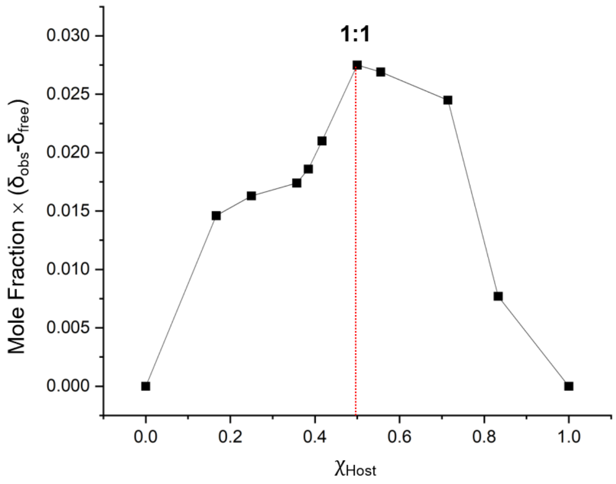

Figure S40. Job plot showing a peak maximum was reached around 0.5, corresponding to the formation of a 1:1 host–guest complex between **1** and $NaClO_4$ in $CDCl_3$ at 298 K.

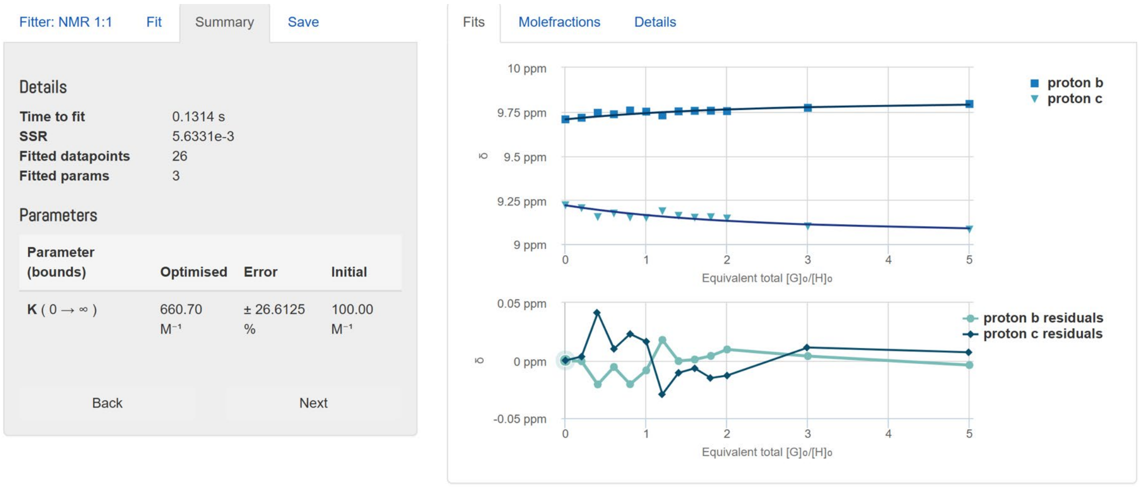

Figure S41. Nonlinear least-square analysis of the ^{1}H NMR binding data corresponding to the formation of **1** ⊃ $NaClO_4$ complex. The data extracted from Fig. S39 were fitted to a 1:1 binding model to give K_a = (6.61 ± 1.76) × 10^2 M^{-1}. The residual distribution is shown below the binding isotherm. All solid lines were obtained from non-linear curve-fitting to a 1:1 binding model using the www.supramolecular.org web applet.

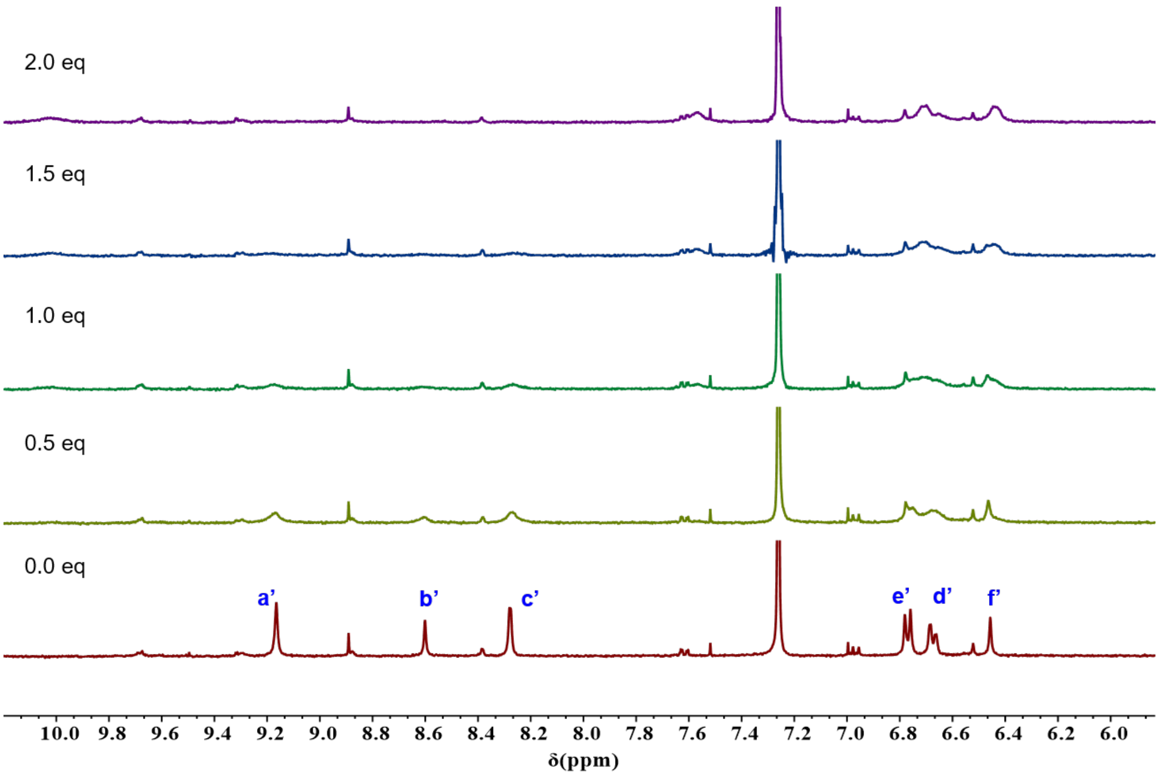

Figure S42. Partially stacked ^{1}H NMR spectra ($CDCl_3$, 400 MHz, 298 K) of UV-pretreated (365 nm) macrocycle **2c** (1.0 mM), titrated with $LiClO_4$ from 0 equiv. to 2.0 equiv.

6. DFT calculations

All density functional theory (DFT) calculations were performed using the Gaussian 09 program package (Revision C.01). Geometries were fully optimized without symmetry constraints at the B3LYP level of theory. The 6-31G(d) basis set was employed for all atoms (C, H, N, and O). Frequency calculations were carried out at the same level of theory to confirm the nature of the stationary points (no imaginary frequencies for minima and one imaginary frequency for transition states). The Gibbs free energy corrections were obtained from frequency calculations at 298.15 K and 1 atm.

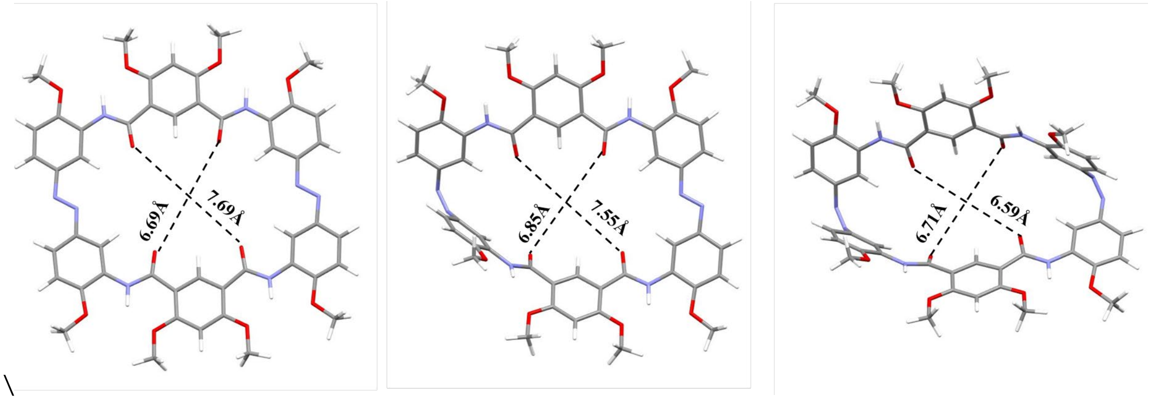

Figure S43. DFT-optimized structures of *E,E*-**2e**, *Z,E*-**2e**, and *Z,Z*-**2e**.

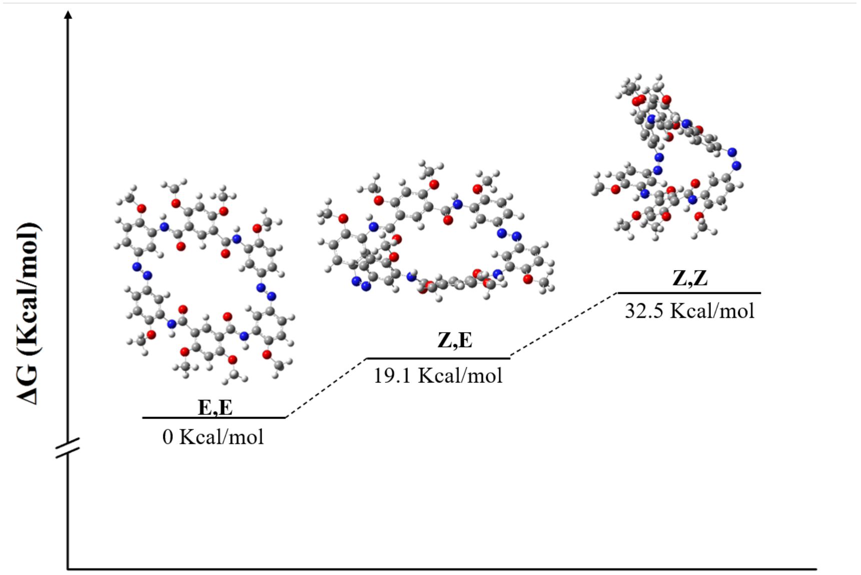

Figure S44. Structures, relative energies of the three stereoisomers of **2e**. The energy is measured in kcal/mol and is compared to the lowest energy conformation of *E,E*, which is set to 0 kcal/mol.

References

[1] Ye, Z.; Yang, Z.; Wang, L.; Chen, L.; Cai, Y.; Deng, P.; Feng, W.; Li, X.; Yuan, L. A Dynamic Hydrogen-Bonded Azo-Macrocycle for Precisely Photo-Controlled Molecular Encapsulation and Release, *Angew. Chem. Int. Ed.* **2019**, *58*, 12519–12523.

[2] Yuan, L.; Sanford, A. R.; Feng, W.; Zhang, A.; Zhu, J.; Zeng, H.; Yamato, K.; Li, M.; Ferguson, J. S.; Gong, B. Synthesis of Crescent Aromatic Oligoamides, *J. Org. Chem.* **2005**, *70*, 10660–10669.

[3] Xie, Y.; Huang, B.; Yu, K.; Shi, F.; Xu, W. Discovery of a Series of Novel Compounds with Moderate Anti-Avian H5N1 Influenza Virus Activity in Chick Embryo, *Med. Chem. Res.* **2013**, *22*, 3485–3496.

[4] Heo, N. J.; Oh, J. H.; Li, A.; Lee, K.; He, Q.; Sessler, J. L.; Kim, S. K. Ion Pair Extractant Selective for LiCl and LiBr, *Chem. Sci.* **2024**, *15*, 13958–13965.
